# Structure of the *Cladosporium fulvum* Avr4 effector in complex with (GlcNAc)_6_ reveals the ligand-binding mechanism and uncouples its intrinsic function from recognition by the Cf-4 resistance protein

**DOI:** 10.1371/journal.ppat.1007263

**Published:** 2018-08-27

**Authors:** Nicholas K. Hurlburt, Li-Hung Chen, Ioannis Stergiopoulos, Andrew J. Fisher

**Affiliations:** 1 Department of Chemistry, University of California, Davis, Davis, California, United States of America; 2 Department of Plant Pathology, University of California, Davis, Davis, California, United States of America; 3 Department of Molecular and Cellular Biology, University of California, Davis, Davis, California, United States of America; Nanjing Agricultural University, CHINA

## Abstract

Effectors are microbial-derived secreted proteins with an essential function in modulating host immunity during infections. *Cf*Avr4, an effector protein from the tomato pathogen *Cladosporium fulvum* and the founding member of a fungal effector family, promotes parasitism through binding fungal chitin and protecting it from chitinases. Binding of Avr4 to chitin is mediated by a carbohydrate-binding module of family 14 (CBM14), an abundant CBM across all domains of life. To date, the structural basis of chitin-binding by Avr4 effector proteins and of recognition by the cognate Cf-4 plant immune receptor are still poorly understood. Using X-ray crystallography, we solved the crystal structure of *Cf*Avr4 in complex with chitohexaose [(GlcNAc)_6_] at 1.95Å resolution. This is the first co-crystal structure of a CBM14 protein together with its ligand that further reveals the molecular mechanism of (GlcNAc)_6_ binding by Avr4 effector proteins and CBM14 family members in general. The structure showed that two molecules of *Cf*Avr4 interact through the ligand and form a three-dimensional molecular sandwich that encapsulates two (GlcNAc)_6_ molecules within the dimeric assembly. Contrary to previous assumptions made with other CBM14 members, the chitohexaose-binding domain (ChBD) extends to the entire length of *Cf*Avr4 with the reducing end of (GlcNAc)_6_ positioned near the N-terminus and the non-reducing end at the C-terminus. Site-directed mutagenesis of residues interacting with (GlcNAc)_6_ enabled the elucidation of the precise topography and amino acid composition of Avr4’s ChBD and further showed that these residues do not individually mediate the recognition of *Cf*Avr4 by the Cf-4 immune receptor. Instead, the studies highlighted the dependency of Cf-4-mediated recognition on *Cf*Avr4’s stability and resistance against proteolysis in the leaf apoplast, and provided the evidence for structurally separating intrinsic function from immune receptor recognition in this effector family.

## Author summary

Microbes mobilize an array of secreted effectors to manipulate their hosts during infections, whereas in response, hosts utilize cognate immune receptors to perceive effectors and mount a defense. To date, the structural basis of effector function and recognition by immune receptors are still poorly understood. Here we present the crystal structure in complex with chitohexaose of *Cf*Avr4, a CBM14 lectin and the founding member of a fungal effector family that binds and protects chitin in fungal cell-walls from chitinases. This is the first structure of a CBM14 protein to be co-crystalized with its ligand that further reveals how Avr4 effectors function. Specifically, by leveraging structural and functional data, we elucidate the molecular basis for ligand-binding by *Cf*Avr4 and show that two effector molecules are brought together through the ligand to form a sandwich structure that laminates two chitohexaose molecules within the dimeric assembly. We further show that recognition of *Cf*Avr4 by the cognate Cf-4 immune receptor is not mediated through residues directly interacting with chitohexaose, thereby structurally uncoupling the ligand-binding function of Avr4 from recognition by Cf-4 and challenging early postulations that the broad recognition of Avr4 effectors by Cf-4 stems from perceiving residues implicated in binding their ligand.

## Introduction

Effectors are intriguing and enigmatic proteins deployed by microbes during host-pathogen interactions [1, 2]. Although inhibition of plant immunity during host infection is the main function of these proteins, the manner by which individual effectors perform this task is poorly understood [2]. One of the better understood effectors is *Cf*Avr4, a 135-residue effector protein from the tomato pathogen *Cladosporium fulvum*, which utilizes a carbohydrate-binding module of family 14 (CBM14) to bind chitin present in fungal cell walls and protect it from hydrolysis by plant-derived chitinases during infection [3–5]. To date, functional orthologues of Avr4 have been identified in a number of fungal species within the Dothideomycete class of fungi and beyond, including the tomato pathogen *Pseudocercospora fuligena* [6], the banana pathogen *Pseudocercospora fijiensis* [4], and several others [7]. The majority of Avr4 homologs share a similar cysteine-spacing pattern and contain a distinctive CBM14 domain in their structure, indicating that members of the Avr4 effector family have a conserved role in binding and protecting chitin in fungal cell walls against chitinases [4, 6]. Moreover, biochemical analysis between *Cf*Avr4 and its *Pf*Avr4 homolog from *P*. *fuligena* has shown that the specificity of these proteins extends further into binding the same length chito-oligosaccharide, i.e. (GlcNAc)_6_, suggesting that they share a similar binding-site topography and mechanism of interacting with the ligand [6].

Although protection of chitin seems to be the predominant biological function of the Avr4 family members [4, 6], a molecular-level mechanistic understanding of how these effectors bind to their substrate is currently lacking. In this respect, the presence of a CBM14 module in the structure of Avr4 is likely key to its biological function and interaction with chito-oligomers. CBM14s are short modules of approximately 70 residues that bind explicitly to chitin, a long-chain polymer of β(1–4) linked *N*-acetylglucosamine (GlcNAc) [7]. Currently, only limited information exists as to how CBM14 family members bind to their ligand, but the inclusion of the CBM14 family within the Type C class of CBMs suggests that they lack the extended binding site found in CBM Types A and B [7, 8]. However, an experimental validation of this assumption is currently lacking.

Despite the lack of information regarding the structural basis for ligand-binding by CBM14 proteins, clues to how Avr4 could possibly interact with its ligand were recently provided by the elucidation of the crystal structure of *Pf*Avr4 from *P*. *fuligena* [6]. Like *Cf*Avr4, *Pf*Avr4 utilizes a CBM14 domain to bind specifically to (GlcNAc)_6_ oligomers, while binding to higher molecular weight chitin may be facilitated through positive cooperative protein-protein interactions of *Pf*Avr4 molecules [6, 9]. Although, a co-crystal of *Pf*Avr4 with chito-oligomers was not attainable, the chitohexaose-binding domain (ChBD) of *Pf*Avr4 was predicted to be located to the C-terminus of the protein [6]. The predicted location of the ChBD in *Pf*Avr4 matches to that of *Cf*Avr4, which used NMR titration experiments to identify residues that may interact with chito-oligomers but was unable to resolve its three-dimensional structure [9]. Next to *Pf*Avr4, the only known structures of CBM14 members are those of tachycitin [10], a small antimicrobial protein from horseshow crab, Der p 23 [11], an allergen from the dust mite *Dermatophagoides pteronyssinus*, and the ChBD of the human chitotriosidase CHIT1 (ChBD_CHIT1_) [12, 13]. All four structures share a common core containing two β-sheets in a distorted β-sandwich arrangement, a seemingly common fold among CBMs [14].

Next to a conserved biological function, another unexpected commonality shared among several Avr4 family members is their ability to elicit a hypersensitive response (HR) in the presence of the cognate Cf-4 resistance protein from tomato [4, 6]. As most Avr4 orthologues share little sequence similarity, it was hypothesized that the indispensability of the CBM14 domain for the function of the protein would make it a prime target for recognition by Cf-4 [4]. However, structure-function analysis with *Pf*Avr4 has shown that point mutations in residues within the predicted ChBD of the protein do not abolish recognition by Cf-4, suggesting that amino acids that directly interact with (GlcNAc)_6_ do not form part of *Cf*Avr4’s epitope that is recognized by Cf-4 [6]. However, a shortcoming of the studies on *Pf*Avr4 was that the analysis was based on a predictive model of Avr4’s ChBD instead of structural information regarding the actual carbohydrate-protein interaction.

Here, we report the crystal structure of *Cf*Avr4 bound to (GlcNAc)_6_, thereby accurately now defining the architecture and amino acid composition of the ChBD and elucidating the underlying molecular mechanism employed by Avr4 family members to bind (GlcNAc)_6_ oligosaccharides. The *Cf*Avr4-(GlcNAc)_6_ complex showed that, contrary to previous assessments, the ChBD of *Cf*Avr4 extents nearly to the entire length of the protein, which in the presence of the ligand forms a dimeric assembly that laminates two (GlcNAc)_6_ oligosaccharides within its structure. Subsequent detailed functional profiling of residues involved in binding (GlcNAc)_6_ has further enabled us to quantify their individual contribution to binding affinity, thereby identifying the ones that are most critical to ligand-binding. Further on, by leveraging structural and functional data we reassessed whether the pleiotropic recognition of Avr4 effectors by Cf-4 is based on the perception of residues that directly interact with (GlcNAc)_6_ and established that such residues are not targets for recognition by Cf-4, thus provided now strong evidence for structurally separating the ligand-binding function in the Avr4 effector family from recognition by Cf-4.

## Results

### *Cf*Avr4 shares structural similarity with *Pf*Avr4 and other CBM14 family members

Our previous crystal structure of *Pf*Avr4 proved recalcitrant to structures complexed with chito-oligosaccharides [6]. Consequently, we undertook crystallization of *Cf*Avr4, which has a 10-fold higher binding affinity for (GlcNAc)_6_ than *P*fAvr4, and were successful in obtaining crystals of *Cf*Avr4 in complex with (GlcNAc)_6_. *Cf*Avr4 bound in a 1:1 stoichiometric ratio with the (GlcNAc)_6_ ligand and the final structure of the *Cf*Avr4-(GlcNAc)_6_ complex was solved at 1.95Å resolution, with R-factor and R-free values of 16.7% and 21.4%, respectively (S1 Table).

**Fig 1:**
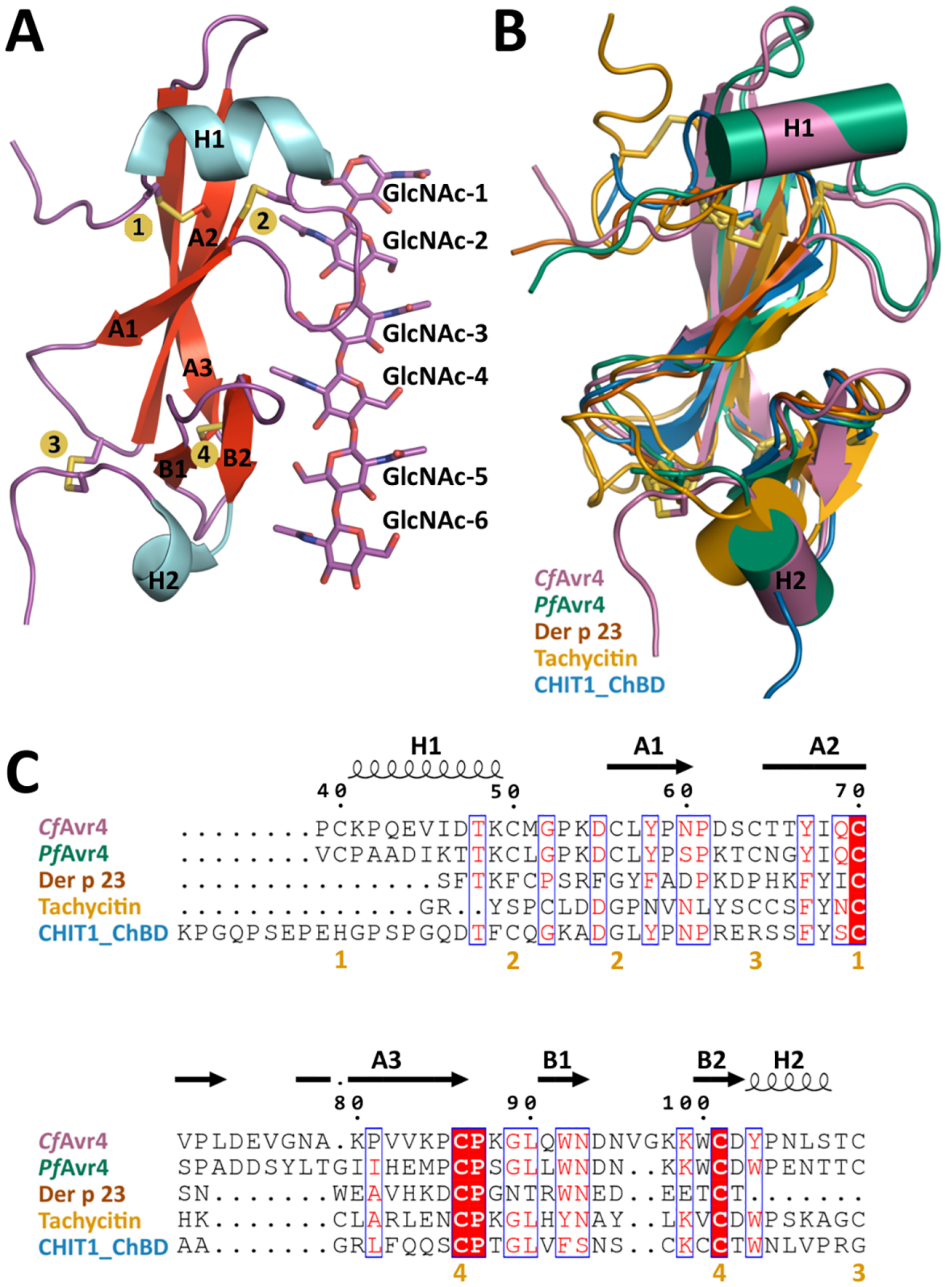
The tertiary and secondary structure of *Cf*Avr4. **(A)** Shown is a *Cf*Avr4 monomer bound to the corresponding (GlcNAc)_6_ molecule (sticks). GlcNAc-1 represents the reducing end of the hexasaccharide sugar. The secondary structure elements are labeled as well as the four disulfide bonds (numbers highlighted in yellow). **(B)** The five members of CBM14 with known structures are superimposed. *Cf*Avr4 (PDB Id: 6BN0) is shown in purple, *Pf*Avr4 (PDB Id: 4Z4A) in green, Der p 23 (PDB Id: 4ZCE) in brown, tachycitin (PDB Id: 1DQC) in yellow, and ChBD_CHIT1_ (PDB Id: 5HBF) in blue. **(C)** Sequence alignment of the five CBM14 family members with known structures. The structural elements of *Cf*Avr4 are shown. Mostly conserved or similar residues are shown in red with the blue boxes. Completely conserved residues are highlighted in red boxes. The disulfide bonds of *Cf*Avr4 are signified by the yellow numbers under the sequences.

The X-ray crystal structure of the *Cf*Avr4 monomer spans residues Gln35-Thr113 and is composed of an N-terminal α-helix (H1), a distorted β-sandwich fold formed by a central β-sheet (A) with three anti-parallel β-strands (A1, A2, and A3), a small β-sheet (B) of two anti-parallel β-strands (B4 and B5), and a short C-terminal α-helix (H2) (Fig 1A). Nearly 47% of the structure is organized into α/β secondary structure with the remaining 53% residing in highly ordered loops. The structure clearly shows four disulfide bonds that match the previously determined disulfide pairs of Cys40-Cys70, Cys50-Cys56, Cys64-Cys109, and Cys86-Cys101 [6, 15]. A comparison with the *Pf*Avr4 structure shows that the two proteins share a similar fold, with the two structures aligning with a root-mean-squared deviation (RMSD) of 0.794 Å over 53 α-carbons (Fig 1B and S1A Fig). The only notable discrepancy resides in the proteins’ C-terminus, which in both is comprised of a β-sheet B and an α-helix H2, but *Cf*Avr4 has a two-residue insertion extending the loop connecting the β-strands B1 and B2 (Fig 1C). A search using the DaliLite server [16] revealed that despite the lack of similarity at the amino acid level, *Cf*Avr4 also shares significant structural homology to the CBM14 family members tachycitin (PDB Id: 1DQC), Der p 23 (PDB Id: 4ZCE), and ChBD_CHIT1_ (PDB Id: 5HBF), aligning to these proteins at an RMSD of 2.019 Å, 0.699 Å, and 0.686 Å, over 36, 16, and 38 α-carbons, respectively (Fig 1B and S1B–S1D Fig). However, all three CBM14 proteins lack the N-terminal helix and the large extended loop connecting β-strands A2 and A3 present in *Cf*Avr4 and *Pf*Avr4, plus Der p 23 and ChBD_CHIT1_ also lack the C-terminal helix. Moreover, three of the four disulfide bonds in *Cf*Avr4 are conserved in tachycitin but only two appear in the Der p 23 and the ChBD_CHIT1_ structures (S1B–S1D Fig).

### The *Cf*Avr4-(GlcNAc)_6_ co-crystal structure reveals a dimeric assembly that engulfs the oligosaccharide substrate

**Fig 2:**
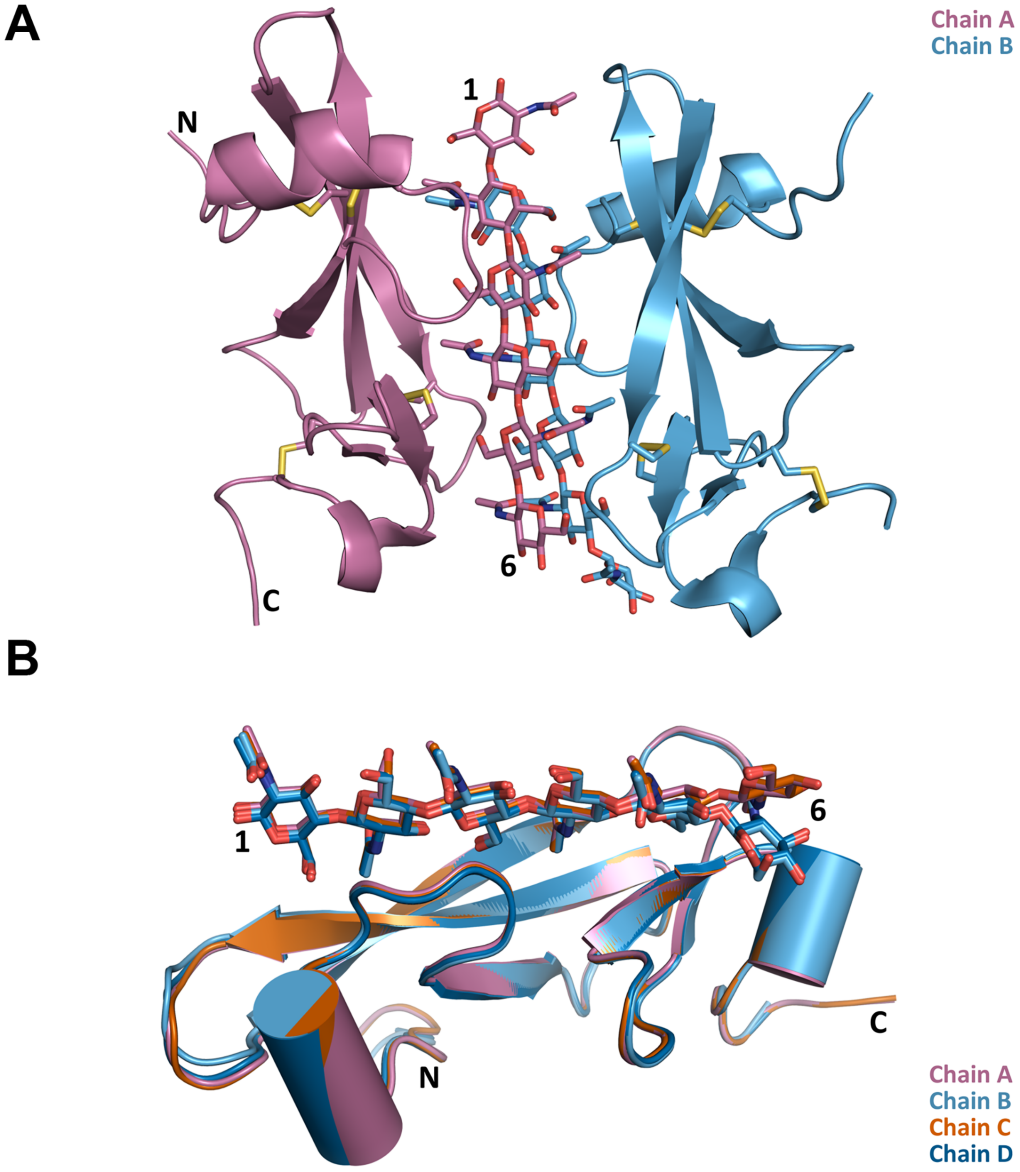
The dimeric *Cf*Avr4-(GlcNAc)_6_ structure. **(A)** Two *Cf*Avr4 monomers shown in purple and blue respectively, bind to two (GlcNAc)_6_ molecules (sticks). The disulfide-bonds are illustrated as yellow-colored sidechains. All dimeric contacts are mediated through the two chitin hexasaccharides. **(B)** Superposition of the four *Cf*Avr4 monomers observed in the crystallographic asymmetric unit. The (GlcNAc)_6_ molecules are shown as stick models. The hexasaccharides line up almost exactly except for GlcNAc-6 on chains B and D, which bend towards the protein. Aligned to chain A, chains B, C, and D have an RMSD of 0.309 Å, 0.099 Å, and 0.265 Å, over 67, 73, and 68 α-carbons, respectively.

*Cf*Avr4 was crystallized with (GlcNAc)_6_ as an asymmetric unit consisting of two *Cf*Avr4 dimers, with each dimer respectively binding to two (GlcNAc)_6_ molecules (S2 Fig and S1 Movie). However, only ~610 Å^2^ of surface area is buried between the two dimers, suggesting that the tetrameric unit is likely crystallographically induced and that the biologically relevant assembly is a dimer, as previously proposed for *Pf*Avr4 [6, 9]. Each dimer consists of two *Cf*Avr4 monomers bound to two parallel (GlcNAc)_6_ molecules stacked on top of each other (Fig 2A, S3 Fig and S2 Movie). A single (GlcNAc)_6_ chain nearly extends along the entire length of the longitudinal axis of each *Cf*Avr4, with the reducing end located near the N-terminus of the protein and the non-reducing end at the C-terminus. Within each dimer, the stacked (GlcNAc)_6_ molecules shift by translation of one sugar ring, with GlcNAc-1 (reducing end) of chain B stacking on top of GlcNAc-2 of chain A and so forth (Fig 2A and S4 Fig). The dimeric assembly creates a 2-fold screw-like rotation of each monomer, such that both sugar and protein are rotated 180° and translated by one sugar unit to create a molecular sandwich that almost entirely encapsulates the parallel-stacked (GlcNAc)_6_ molecules within its structure. Surprisingly, the *Cf*Avr4 dimer interface is completely mediated by carbohydrate interactions and no intermolecular protein-protein interactions are observed across the dimer, suggesting that dimerization is a consequence of ligand binding. When considering individual *Cf*Avr4 monomers in the crystallographic asymmetric unit, all four show nearly identical conformations and (GlcNAc)_6_ interactions (Fig 2B). Aligned to chain A, chains B, C, and D have an RMSD of 0.309 Å, 0.099 Å, and 0.265 Å, over 67, 73, and 68 α-carbons, respectively (Fig 2B). In chains B and D, however, the GlcNAc-6 ring bends toward the protein, deviating from the linearity that is seen in the other two chains.

Interestingly, *Pf*Avr4 has been previously crystalized as a dimer as well [6] but a comparison of the *Cf*Avr4-(GlcNAc)_6_ dimeric assembly to the ligand-free *Pf*Avr4 dimer showed that two dimer arrangements vary substantially (S5 Fig). For instance, in *Pf*Avr4, the dimeric contacts were mostly water-mediated and only three direct hydrogen-bond interactions between monomer chains were detected [6]. In contrast, *Cf*Avr4 dimerization is mediated exclusively by (GlcNAc)_6_, as no direct protein-protein interactions are observed in the *Cf*Avr4-(GlcNAc)_6_ dimeric assembly. Instead, several cross linkages are formed where each monomer of *Cf*Avr4 interact with both (GlcNAc)_6_ molecules to stabilize the dimeric state of the complex. Further comparison of the dimeric assemblies reveals that upon superposition of the A chain monomers of *Pf*Avr4 and *Cf*Avr4, the B subunit of *Cf*Avr4 is shifted out ~6.7Å and rotated ~54° relative to the B subunit of *Pf*Avr4 (S5 Fig). This increased separation between the *Cf*Avr4 monomers is caused by enclosing the ligands at the dimerization interface and creating a space large enough for accommodating two (GlcNAc)_6_ molecules between the monomers.

### The crystal structure of *Cf*Avr4 in complex with (GlcNAc)_6_ elucidates the architecture and amino acid composition of its ChBD

By modelling the *Pf*Avr4 crystal structure on other chitin-binding proteins, we have previously predicted that *Pf*Avr4’s ChBD resides in the C-terminal domain of the protein consisting of residues on β-strands B4 and B5 and their connecting β-hairpin loop. However, its exact topography and composition could not be precisely defined as even after repeated crystallization attempts a *Pf*Avr4-(GlcNAc)_6_ co-crystal was never obtained [6].

**Fig 3:**
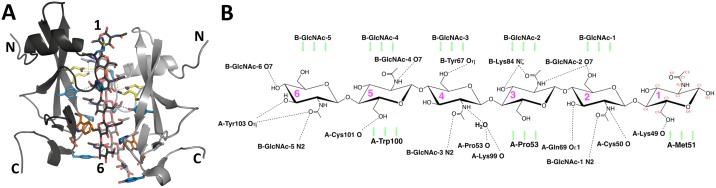
The molecular interactions between *Cf*Avr4 and (GlcNAc)_6_. **(A)** Shown is the *Cf*Avr4 dimer with residues that contact the (GlcNAc)_6_ molecules drawn with stick sidechains. Residues that do not substantially affect binding are in blue, residues significantly affecting binding are in yellow, and residues that abolish binding are in orange. **(B)** Schematic representation of *Cf*Avr4-(GlcNAc)_6_ interactions for the A subunit hexasaccharide. Hydrogen bonds are represented by black dashed lines, van der Waals interactions stacking against the pyranose rings are illustrated by green hashed lines.

Contrary to previous assessments [6, 10, 15], the chitin hexasaccharide is accommodated in a shallow trench across the longitudinal axis of *Cf*Avr4 with the face of the pyranose rings binding to the protein by means of nonpolar and CH-π interactions, and the ring substituents pointing into the protein core and forming hydrogen bonds with both the main chain and side chains (Fig 3A and S4 Fig). The main facial interaction between *Cf*Avr4 and (GlcNAc)_6_ is a CH-π bond between Trp100 and GlcNAc-5. Met51 and Pro53 are also in van der Waals bonding distances of GlcNAc-1 and GlcNAc-3, respectively and contribute to ligand binding. In addition, a number of amino acids in *Cf*Avr4 are also shown to interact with individual sugar ring substituents of the (GlcNAc)_6_ substrate (Fig 3B, S4 Fig and S2 Table). For instance, starting from GlcNAc-1, the C6 hydroxyl of GlcNAc-1 forms a hydrogen bond with the main chain carbonyl oxygen of Lys49. The *N*-acetyl group nitrogen of GlcNAc-2 hydrogen bonds with the main chain carbonyl oxygen of Cys50, while its C3 hydroxyl group hydrogen bonds to the amide oxygen of Gln69. The *N*-acetyl group nitrogen of GlcNAc-4 forms a water-mediated hydrogen bond interaction with the main chain carbonyl oxygens of both Pro53 and Lys99, an association that is observed in all 4 monomers. The C6 hydroxyl of GlcNAc-5 forms a hydrogen bond with the main chain carbonyl of Cys101, whereas the hydroxyl of Tyr103 is also within hydrogen bonding distance to both the GlcNAc-6 *N*-acetyl carbonyl and the C3 hydroxyl. In chain B, GlcNAc-6, which does not stack with a carbohydrate subunit from chain A, bends towards the protein so that its *N*-acetyl group comes within hydrogen bonding distance to the side chain of Asp102 (Fig 3B, S4 Fig and S2 Table). Taken together, the interactions of a *Cf*Avr4 monomer with the (GlcNAc)_6_ molecule is mediated by residues Lys49, Cys50, Met51, Pro53, Lys99, Trp100, Cys101, Asp102, and Tyr103, which collectively form at least two water-mediated and nine direct hydrogen bonds with (GlcNAc)_6_. Of these, only Trp100 (Trp94 in *Pf*Avr4), Asp102 (Asp96 in *Pf*Avr4), and Trp103 (Tyr97 in *Pf*Avr4) have been previously identified as ChBD residues in *Pf*Avr4 [6].

Interestingly, while no direct protein-protein interactions are observed in the *Cf*Avr4-(GlcNAc)_6_ dimeric assembly, there are plenty of sugar-facilitated cross-linkages between the two protein chains (Fig 3B, S4 Fig and S2 Table). For instance, the hydroxyl of Tyr67/A hydrogen bonds to the C6 hydroxyl of the GlcNAc-2/B, whereas the same interaction occurs between Tyr67/B and the GlcNAc-4/A. In a similar way, the amine of Lys84/A hydrogen bonds to the *N*-acetyl carbonyl of GlcNAc-1/B, whereas Lys84/B interacts with the *N*-acetyl carbonyl of GlcNAc-3/A. The amide nitrogen of Gln69/A hydrogen bonds with the C3 hydroxyl of GlcNAc-1/B, while Gln69/B hydrogen bonds to C3 hydroxyl of GlcNAc-2/A. Finally, the hydroxyl of Tyr103/A hydrogen bonds to the *N*-acetyl group of GlcNAc-5/B, but this interaction is not seen between Tyr103/B and the A sugar, as it would have to occur with a GlcNAc-7 unit on the chain A.

### Site-directed mutagenesis identifies residues that significantly contribute to binding affinity to (GlcNAc)_6_

To assess the functional role that each residue in *Cf*Avr4 involved in binding (GlcNAc)_6_ has, we mutated these residues individually and investigated the binding properties via Isothermal Titration Calorimetry (ITC). Specifically, we made the alanine-substitution mutations M51A, P53A, K84A, P87A, W100A, and D102A, and the more conservative mutations Q69N, Y67F and Y103F, to minimize potential protein instability effects. We excluded Lys49 from the mutational analysis since only the main chain of this residue interacts with (GlcNAc)_6_, as well as Cys50 and Cys101 because it has been previously shown that mutating these residues compromises the stability of the protein [9, 15].

ITC experiments determined that WT-*Cf*Avr4 bound to (GlcNAc)_6_ with a dissociation constant (*K*_d_) of 6.73 ± 1.49 μM, a binding enthalpy (Δ*H*) of -38.37 ± 2.96 kJ/mol, and a stoichiometric ratio *n* of 1.09 ± 0.12 (S2 Table and S6 Fig). The parameters remained similar irrespectively of whether or not the purification tag was removed from the protein or whether a reverse titration was run, in which concentrated *Cf*Avr4 was titrated into a dilute solution of (GlcNAc)_6_, with no evidence of allosteric or dimer dissociation (S2 Table and S6 Fig). The values are also in good agreement with previously reported data except for the stoichiometric ratio [9]. Specifically, our ITC experiment resulted in a stoichiometric ratio of 1:1, which agrees with the structure, but contrasts the 2:1 protein:(GlcNAc)_6_ stoichiometry proposed previously [9]. Due to this discrepancy, we took great care to verify the concentration of *Cf*Avr4 using a Bradford assay, and quantitated the carbohydrate concentration [17, 18]. When assaying the ChBD mutants, mutations M51A, P53A, Y67F, P87A, and Y103F did not affect the *K*_d_ substantially and had a small decrease in the Δ*H* magnitude (S2 Table and S6 Fig). However, mutations W100A and D102A abolished all detectable binding to (GlcNAc)_6_, in agreement with the previously reported analogous mutations made in *Pf*Avr4 [6]. Mutations Q69N and K84A both displayed a ~20-fold increase in the *K*_d_ raising it to 130.95 ± 31.47 μM and 120.67 ± 18.77 μM, respectively (S2 Table and S6 Fig).

To determine if the reduced affinity for (GlcNAc)_6_ of the W100A, D102A, Q69N and K84A mutants was biologically meaningful, we evaluated whether they were able to protect germlings of *Trichoderma viride* against hydrolysis from chitinases and compared their protective potency to that of the WT-*Cf*Avr4 (S7 Fig). Similar *in vitro* protection assays were used before to demonstrate and compare the protective properties of *Cf*Avr4, *Pf*Avr4 and other Avr4 family members or mutants thereof [4–6]. As expected, combined addition to pre-germinated germlings of *T*. *viride* of BSA (negative control) with chitinases supplemented with basic β-1,3-glucanases inhibited fungal growth, whereas combined application of the enzyme mixture with the WT-*Cf*Avr4 (positive control) enabled fungal growth and survival (S7 Fig). When examining the ChBD mutants, mutants W100A and D102A that do not exhibit any detectable binding to (GlcNAc)_6_ (S2 Table and S6 Fig), essentially failed to protect the fungal hyphae against chitinases, thus resulting in poor fungal growth that was comparable to that of the BSA control. Mutants Q69N and K84A that exhibit reduced affinity for (GlcNAc)_6_ (S2 Table and S6 Fig), enabled fungal growth to levels comparable to those of the WT-*Cf*Avr4, although at closer inspection of the hyphae many were now seen to bore signs of osmotic injuries such as swollen segments and coagulated cytoplasm (S7 Fig). This indicates that the chitinase treatment had a stronger effect on hyphae treated with the Q69N and K84A mutants as compared to hyphae treated with the WT-*Cf*Avr4 that remained morphologically intact. Collectively, results from the protection assays show that mutations in the ChBD of *Cf*Avr4 that decrease or abolish its affinity for (GlcNAc)_6_ also reduce the protein’s ability to protect fungal germlings against chitinases and thus perform its biological function.

A previous NMR study of *Cf*Avr4, while unable to determine the structure, showed amide (^1^HN) backbone chemical shifts upon addition of (GlcNAc)_3_ that were assigned to Asn93, Asp94, Asn95, (NDN motif), Asp102, and Tyr103 [9]. The structure of *Cf*Avr4 in complex with (GlcNAc)_6_ confirms that Asp102 and Tyr103 directly interact with the ligand. In contrast, the NDN motif is located on the B4-B5 β-hairpin loop and is pointing away from the binding site, where it is at a distance of more than 10Å from the hexasaccharide (S8 Fig). To address this discrepancy between the NMR and crystallographic data, we made mutations N93A, D94A and N95A, and characterized their affinity for (GlcNAc)_6_ using ITC (S2 Table). Surprisingly, all three mutations affected the thermodynamic parameters that were indicative of lower affinity for the hexasaccharide, as they increased the *K*_d_ by a factor of six (N93A), five (D94A) and three (N95A) (S2 Table). One of the characteristics of CH-π interactions, such as observed between Trp100 and GlcNAc-5, is that they are very sensitive to the electronic structure of the aromatic residues involved in binding [19]. It is thus likely that the nearby NDN motif helps to create a proper electronic environment to facilitate binding, while also providing structural integrity to the C-terminal domain containing Trp100 (see below). The amide side chain of Asn93 is 4.0 Å from the indole ring nitrogen of Trp100, and further hydrogen bonds to both side chain and main chain of Asn95, thus stabilizing the NDN loop. Therefore, the binding of the hexasaccharide to Trp100 could shift this loop, resulting in the observed backbone NMR chemical shifts [9].

### The recognition of *Cf*Avr4 by Cf-4 is not mediated directly by residues that interact with (GlcNAc)_6_

We have previously determined that residues in *Pf*Avr4 that are critical to binding (GlcNAc)_6_ do not individually have an effect on *Pf*Avr4’s interaction with Cf-4, as alanine substitution of these residues yields avirulent forms of the protein that elicit a Cf-4-mediated HR. Instead, a strong correlation between receptor activation and Avr4 stability was observed, as ChBD mutants that escape detection by Cf-4 are unstable proteins that are susceptible to proteolytic cleavage in the protease-rich environment of the leaf apoplast [6]. These observations prompt us to suggest that the ligand-binding function of Avr4 is structurally distinct or does not fully overlap with the property of recognition by Cf-4. This is important because it alluded that the molecular basis for the pleiotropic recognition of core effector proteins by single immune receptors is not based on the perception of individual amino acids that define the effectors intrinsic function, as previously hypothesized [4].

**Fig 4:**
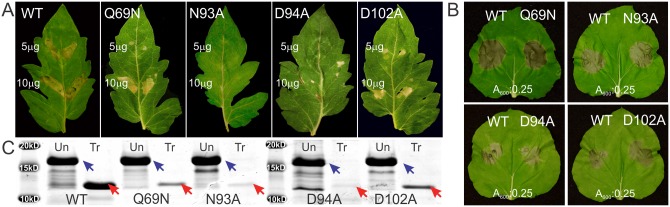
Recognition by the Cf-4 immune receptor and susceptibility to proteolytic degradation of the WT-*Cf*Avr4 and selected ChBD and (GlcNAc)_6_-binding mutants. **(A)** Protein infiltrations of the WT-*Cf*Avr4 (WT) into tomato leaves of cv. Purdue 135 (+Cf-4) induces a strong hypersensitive response (HR) in the infiltrated leaf sectors at both protein concentrations tested. However, *Cf*Avr4 mutants Q69N, D102A only partially trigger an HR and N93A, D94A do not, as seen by the reduced intensity of necrosis. All other ChBD or (GlcNAc)_6_-binding mutants of *Cf*Avr4 tested (S2 Table) induced the same as the WT-*Cf*Avr4 intensity of HR (S9A Fig). Infiltrations were performed on both the left and right sides of the leaf, and necrosis was evaluated 5 days post-infiltration (dpi). **(B)** Transient co-expression with Cf-4 of the WT-*Cf*Avr4 (WT) and the Q69N, N93A, D94A and D102A mutants into leaves of *Nicotiana benthamiana* using an *Agrobacterium tumefaciens* transient transformation assay, induces, in all cases, a strong and similar in intensity HR. All other mutants are shown in S9B Fig. In all cases, co-infiltrations of Cf-4 with the WT-*Cf*Avr4 were done on the left-hand side of the leaf, whereas co-infiltrations of Cf-4 with the mutant was done on the right-hand side of the leaf. Pictures were taken at 7 dpi. **(C)** Treatment of the WT-*Cf*Avr4 and Q69N, N93A, D94A, and D102A mutants with 500 ng/μl subtilisin, digests the original full-length protein (blue arrows) to a smaller product that corresponds to the true mature form of *Cf*Avr4 (red arrows). The treatments show that all mutants are vulnerable to proteolytic degradation, as evidenced by the decreased intensity of the mature-form band (red arrows), with mutants N93A and D94A being more susceptible to degradation.

The elucidation of the precise structure and amino acid composition of *Cf*Avr4’s ChBD enabled us to address this postulation with higher accuracy and reexamine whether recognition of Avr4 by Cf-4 is mediated through residues directly interacting with (GlcNAc)_6_. Therefore, we assessed the ability of Cf-4 to mount an HR upon perception of the WT-*Cf*Avr4 and the ChBD mutants (S2 Table). We also assessed mutants N93A, D94A, N95A, as the NDN motif indirectly affects the protein’s affinity for (GlcNAc)_6_. The HR-inducing properties of the effector variants was initially examined by infiltrations of the purified proteins into tomato leaves of cv Purdue 135 (+ Cf-4) and cv Moneymaker (–Cf-4) at concentrations of 5 μg/ml and 10 μg/ml (Fig 4A, S9A Fig and S2 Table). When infiltrated into the leaves of cv Purdue, the WT-*Cf*Avr4 and mutants M51A, P53A, Y67F, K84A, P87A, N95A, W100A and Y103F, all elicited a strong and equal in intensity HR at 5 days post-infiltration (dpi). In contrast, mutants Q69N and D102A elicited an HR at infiltrations with 10 μg/ml but only a weak response at 5 μg/ml, whereas mutants N93A and D94A failed to elicit an HR at both concentrations tested (Fig 4A, S9A Fig and S2 Table). As expected, none of the mutants or the WT-*Cf*Avr4 induced an HR when infiltrated into leaves of cv Moneymaker (S9A Fig). Collectively, these results suggest that residues Gln69, Asn93, Asp94 and Asp102 could be direct targets of recognition by Cf-4 or, alternatively, that they are critical to protein stability and resistance of the protein against proteolytic degradation in the leaf apoplast.

To discriminate between these two possibilities, we next transiently co-expressed Cf-4 with WT-*Cf*Avr4 or its individual mutant alleles into leaves of *Nicotiana benthamiana*, using an *Agrobacterium tumefaciens*-mediated transformation assay (ATTA) [20]. Cf-4:effector co-infiltrations at cell densities of A_600_0.5:A_600_1.0 (0.5:1 ratio), A_600_0.5:A_600_0.5 (1:1 ratio), and A_600_0.5:A_600_0.25 (2:1 ratio) all induced an HR in the infiltrated leaf sectors thus resolving that none of our mutants can effectively escape recognition by Cf-4 when present in sufficient amounts in the leaf apoplast (Fig 4B, S9B Fig and S2 Table). We next compared the resilience of the WT-*Cf*Avr4 and of the Q69N, N93A, D94A and D102A mutants against proteolytic degradation by subtilisin. We additionally included in these assays mutants K84A and W100A as, although they trigger a full and equal in intensity HR as the WT-*Cf*Avr4 (S9 Fig), they nonetheless exhibit less affinity for (GlcNAc)_6_ (S6 Fig and S2 Table). In all cases, treatment with subtilisin resulted in rapid cleavage reducing the full-length protein to the true mature form of *Cf*Avr4 [3, 6] (Fig 4C and S10 Fig). The protease assay showed that with the exception of WT-*Cf*Avr4 and the K84A and W100A mutants (S10 Fig and S2 Table), all other mutants are susceptible to further proteolytic degradation, with mutants N93A and D94A being more so than mutants Q69N and D102A, evidenced by the almost complete disappearance of the band corresponding to the mature *Cf*Avr4 for the N93A and D94A mutants and the decreased intensity of this band for the Q69N and D102A mutants, as compared to the WT-*Cf*Avr4 (Fig 4C). The vulnerability of the mutants to proteolytic degradation is inversely correlated with their ability to elicit a Cf-4 mediated HR (Fig 4B and S2 Table), thus indicating that residues Gln69, Asn93, Asp94 and Asp102 make important contributions to protein stability but they likely do not mediate a direct interaction with Cf-4. Conversely the W100A and K84A mutants, which also show reduced affinity for (GlcNAc)_6_ (S6 Fig and S2 Table) but elicited a full HR in tomato and *N*. *benthamiana* (S9 Fig), are as resilient to proteolysis as the WT-*Cf*Avr4, evidenced by the equal in intensity band corresponding to the mature *Cf*Avr4 obtained in the subtilisin assay for the three proteins (S10 Fig and S2 Table). Taken together, these results indicate that mutations in residues within the ChBD that decrease or abolish the protein’s affinity for (GlcNAc)_6_ do not individually affect recognition by Cf-4 if they do not perturb the stability of the protein.

## Discussion

During the past two decades, a great deal of effort has been placed towards deciphering the molecular determinants that define the structural basis of protein–carbohydrate interactions. Such interactions are ubiquitous in nature and at the heart of diverse biological processes of profound importance to human health, plant growth, and microbial disease [8]. Although CBMs may interact with their oligosaccharide in various ways, generally they do not undergo conformational changes when binding to their ligands. Instead the tertiary structure provides a platform for substrate-binding. Binding-site topography is thus key to their binding mode and can be very diverse, ranging from planar surfaces with aromatic residues that stack against the pyranose rings of polysaccharides (Type A CBMs), to grooves or clefts that contain both aromatic and hydrogen-bonding interactions that accommodate long polysaccharide chains (Type B CBMs), and small pockets that bind short oligosaccharide ligands (Type C CBMs) [8]. A recent refinement of these classes further proposed that Type B CBMs bind glycan chains internally (endo-type), whereas Type C modules interact with short mono-, di-, tri-saccharides or the termini of glycans (exo-type) [21].

To date, only limited information exists on how CBM14 family members bind chito-oligomers, although it is assumed that they would exhibit the structural and functional characteristics of Type C lectins [7, 8]. The assumption mostly stems from indirect evidence and observations that representative members of this family interact with small oligosaccharide ligands, yet the structural basis of this interaction was so far unknown. The structural characterization of *Cf*Avr4 and of its mode of interaction with (GlcNAc)_6_ now provides experimental support that Avr4 may be unique in the CBM14 family in that it has an extended ChBD and can be classified rather as a Type B CBM instead of Type C. This is supported by the fact that Avr4 binds longer polysaccharide chains, whereas its mode of interaction with the substrate is mediated through aromatic residues (i.e. Trp100) and numerous hydrogen bonds with both side chains and main chains. Furthermore, unlike the other structurally-characterized CBM14 family members, *Cf*Avr4 has an additional N-terminal α-helix and an extended loop connecting the α-helix to the first β-strand. The main chain of this loop hydrogen-bonds to (GlcNAc)_6_ (Cys50 to GlcNAc-2) and contains the Cys50-Cys56 disulfide bond along with residues Met51 and Pro53 that stack against the pyranose rings of GlcNAc-1 and GlcNAc-3, respectively. Notably, although mutating each side chain individually only slightly reduces the affinity for (GlcNAc)_6_, the overall additive effect would be increased. These interactions, along with residues Gln69 and Lys84 that greatly affect binding, are not conserved in tachycitin, Der p 23, and ChBD_CHIT1_, which are thus likely to have a smaller ChBD that is more similar to the hevein fold and to bind shorter oligosaccharides in par with other Type C CBMs.

Next to Avr4, the only information available on the ligand-binding mechanism of a CBM14 family member with a known structure is the ligand-free structure of ChBD of the human chitotriosidase CHIT1 (ChBD_CHIT1_) [12, 13]. *Cf*Avr4 and ChBD_CHIT1_ share a similar overall fold but the residues that dictate the binding affinity of the two proteins for their ligand are different. For instance, the residues in *Cf*Avr4 essential for binding (GlcNAc)_6_, Trp100 and Asp102, are replaced by cysteine (Cys462) and threonine (Thr464) in the ChBD_CHIT1_ structure (S1D Fig). In ChBD_CHIT1_, residues Pro451, Leu454, and Trp465 that were identified as crucial for chitin binding [12] are conserved in *Cf*Avr4, aligning to Pro87, Leu90, and Tyr103, respectively. However, although Pro87 and Leu90 sit on the loop connecting the two β-sheets and point toward the ligand binding site suggesting that they will have a role in binding (GlcNAc)_6_, the P87A mutation does not have an effect on the binding thermodynamics of *Cf*Avr4, whereas Leu90 is not within binding distance to (GlcNAc)_6_, as the closest contact is 3.3Å between the terminal methyl of the sidechain of chain A and the C6 hydroxyl of GlcNAc-4 of chain B. Interestingly, ChBD_CHIT1_ binding assays showed that there was no thermodynamic effect on binding when increasing the degree of polymerization beyond the disaccharide of GlcNAc, whereas, Avr4 showed a greatly decreased *K*_d_ and more negative Δ*H* upon an increase to the degree of polymerization of the oligosaccharide. This may indicate a very different binding mechanism between the two ChBDs of the same CBM14 family, which have a conserved fold, or the difference may highlight the vastly different nature of the proteins. CHIT1 is a chitinase with distinct catalytic and ChBD domains and has been implicated in the immune response in humans, whereas Avr4 is a fungal lectin protecting the fungus from plant chitinases. The Avr4 protein most likely needs to bind its ligand with a higher affinity than CHIT1.

The structure of the *Cf*Avr4-(GlcNAc)_6_ complex further showed that two molecules of *Cf*Avr4 can be joined through the ligand to form a sandwich structure that laminates two (GlcNAc)_6_ molecules within the dimeric assembly. This mechanism of ligand-mediated dimerization, to some extent, appears to be unique among carbohydrate-binding proteins, as in most cases dimeric binding involves two molecules of a CBM interacting with the same molecule of the ligand [22–25]. Although it is conceivable that such a dimeric assembly of Avr4 could create a sheltered environment for (GlcNAc)_6_, what cannot be determined is whether it is biologically relevant in terms of how the protein interacts with the network of chitin microfibrils present in the fungal cell wall that more accurately represents the biological substrate of Avr4 under *in vivo* conditions. The cell wall of fungi consists mainly of β-1,3- and β-1,6 glucans cross-linked to randomly oriented microfibrils of chitin, mannans, and glycoproteins that collectively form a three-dimensional layered structure in which glucans and chitin are most frequently positioned closer to the plasma membrane and are overlaid by mannans and glycoproteins [26, 27]. Chitin microfibrils in the fungal cell wall are mainly found in the form of randomly oriented short microcrystalline rodlets and to a lesser extent as a network of longer interlaced microfibrils [28, 29]. Such an arrangement of a tightly knitted network of chitin would seem to preclude the dimer formation that is seen in the crystal structure. Since, Avr4 binds (GlcNAc)_6_ in a 1:1 stoichiometric ratio, it is plausible that the protein rests as a monomer on the solvent-exposed surface of these microfibrils, thus creating a protective layer against endochitinases. If the case, it suggests that Avr4 may interact differently with free in solution chito-oligosaccharides as compared with chitin fixed in microfibrils. Conditional dimer formation has also been observed in the wheat germ agglutinin (WGA), a chitin binding-lectin with a hevein-like fold which, depending on the solution conditions, forms weak non-obligate and transient homodimers. Specifically, it is shown that although dimerization enables WGA to maximize its ligand binding affinity, the monomers exhibit significant binding affinity as well, thus making the formation of the dimers less mandatory for the function of the protein [30]. Another possibility is that binding of the Avr4 monomer to the chitin aggregate may lift a chitin chain from a microfibril, thus loosening the structure and enabling another monomer to grab hold of the opposite strand, thereby capping the ends of it that would otherwise be accessible to exochitinases. This mode of interaction is somewhat analogous to the model proposed for the enzymatic decrystallization of cellulose by celluloses, in which case the CBM appended to the catalytic domain functions as a wedge that lifts cellulose chains from the cellulose network [31, 32]. It should be noted that, depending on the orientation and hydrogen-bonding pattern of its GlcNAc chains, crystalline chitin assembles in nature into mainly three allomorphic forms known as α-, β- or γ-chitin. α-chitin forms antiparallel chains of GlcNAc and is most commonly found in crustaceans, insects, and the cell walls of fungi [33, 34], whereas β-chitin forms parallel chains of GlcNAc and is found mainly in diatoms and cephalopods [35–37]. Lastly, γ-chitin forms a mixture of parallel and antiparallel chains of GlcNAc and is found mainly in cocoon fibers of the *Ptinus* beetle and the stomach of the Loligo squid [38, 39]. Despite the differential orientation of the GlcNAc chains in the three crystalline structures of chitin, they all share a similar stacking pattern of these chains, which is similar to the oligosaccharide stacking observed in the *Cf*Avr4-(GlcNAc)_6_ complex (S11 Fig). This suggests that Avr4 is likely able to bind all three forms of crystalline chitin, which might explain the broad distribution of CBM14 proteins across nearly all domains of life and their involvement in various biological processes [7].

Our previous work on *Pf*Avr4 led us to hypothesize that the ligand-binding function of Avr4 is structurally distinct or does not fully overlap with the property of recognition by Cf-4 [6]. Instead, a strong correlation between receptor activation and Avr4 stability was observed, as ChBD mutants that evade recognition by Cf-4 embody unstable proteins that are susceptible to proteolytic cleavage in the protease-rich environment of the leaf apoplast [6]. The structural determination of the *Cf*Avr4-(GlcNAc)_6_ complex and the elucidation of the precise topography and amino acid composition of *Cf*Avr4’s ChBD enabled us to readdress this postulation now with accuracy and examine whether individual residues that directly interact with (GlcNAc)_6_ are targets for recognition by Cf-4. Our results corroborated the previous findings with *Pf*Avr4, as site-directed mutagenesis of residues in *Cf*Avr4’s ChBD yielded effector mutants that are able to trigger a Cf-4 mediated HR when present at sufficient amounts in the leaf apoplast. The studies further highlighted the dependency of Cf-4-mediated HR on *Cf*Avr4’s stability and resistance against proteolysis in the leaf apoplast, as an inverse correlation exists between the intensity of the HR induced by the mutants when infiltrated into tomato leaves of cv. Purdue 135 (+Cf-4) and their susceptibility to subtilisin. These results are also in agreement with early studies showing that race 4 field isolates of *C*. *fulvum* produce unstable and protease sensitive isoforms of *Cf*Avr4 as a means of evading recognition by Cf-4 [3, 15]. Collectively, these studies emphasize the importance for recognition by Cf-4 of a stable tertiary structure of Avr4 and challenge early postulations that the broad recognition of Avr4 effectors by Cf-4 stems from perceiving residues implicated in binding (GlcNAc)_6_. Instead, we hypothesize that immune receptors like Cf-4 could have exploited, during evolution, the need of apoplastic effectors to adopt a well-ordered stable structure in order to withstand proteolytic attack during infections in leaf apoplast, thus perceiving globular fold properties of Avr4. However, it was previously shown that Pro87, a conserved residue among Avr4 effectors, is essential for the Cf-4-mediated HR, as mutating this amino acid to an arginine resulted in a loss of recognition [40]. Our studies show that Pro87 is in close proximity to the ligand and mutating it to alanine results in minimal effect in ligand binding and no effect on Cf-4 recognition, thus corroborating previous finding with *Pf*Avr4 [6]. This suggests that it is likely not the proline that is essential but a small aliphatic residue that is required in this position in order to ensure the structural integrity of the protein.

## Materials and methods

### Materials

Chitin hexasaccharide was purchased from Megazyme, Inc (Dublin, Ireland). Concentration of WT Avr4 was determined using the theoretical ϵ_280_(Avr4_WT) = 17460 M^-1^ cm^-1^, which was similar to the calculated ϵ_280_ determined from a Bradford Assay. Mutants of Avr4 that included aromatic residues had extinction coefficients different from the WT protein and those values, and that of the WT protein, were determined using the *ExPASy* server [18].

### Determination of chitin hexasaccharide concentration

A stock solution of the monomer of *N*-acetylglucosamine was prepared by dissolving the dry powder in a buffer containing 10 mM Bis-Tris, pH 6.5, 100 mM NaCl to a concentration of 0.100 g/L using analytic techniques. Standard solution of 0, 0.010, 0.025, 0.050, 0.075 g/L were prepared by serial dilutions. Using glass test tubes and pipets, 100 μL of each standard was mixed with 300μL of concentrated sulfuric acid and vortexed for 10 sec to mix. Solutions were incubated at RT for 10 min and then placed on ice to stop the reaction. Solutions had a maximum absorbance at 322 nm and the concentration curve was determined using the absorbance of each standard at this wavelength. Analytically prepared solution of the di- and tri-saccharide scaled linearly to the calibration curve. To determine the concentration of the chitin hexasaccharide solutions used in ITC, the solutions were diluted into the dynamic range of the calibration curve (S12 Fig) and subjected to the above procedure.

### *Cf*Avr4 expression and purification

The part of the *Cf*Avr4 gene that encodes for the true mature form of the protein (i.e. Lys30-Gln115) [3] was cloned into a modified pCDG-duet-1 vector containing two 6x His tags and a rhinovirus 3C protease cleavage site immediately N-terminal of the MCS1 using the SalI and NotI restriction sites. The vector was transformed into *Escherichia coli* Rosetta-gami B cells (Novagen) to facilitate formation of disulfide bonds. For expression, 1L cultures were grown at 37°C until OD_600_ = 0.5–0.7 and then cooled to 15°C. Cultures were induced with IPTG at a concentration of 1mM and allowed to express for 18–24 hours.

For purification, cells were resuspended in buffer containing 50mM Tris:HCl, pH 8.0, 300mM NaCl, and 5mM imidazole. Cells were lysed using a microfluidizer and the lysate was cleared by centrifugation (39,000*xg* for 45 min). Cleared lysate was loaded on a 1mL HiFliQ-NiNTA (Anatrace). The column was washed with 10 column volumes (CV) of lysis buffer containing 30mM imidazole followed by a gradient wash, which increased the imidazole concentration to 60mM over 20CV, then washed with an additional 10CV of buffer containing 60mM imidazole. Protein was eluted from the column using lysis buffer containing 300mM imidazole. Collected protein fractions were concentrated to 1/5^th^ original volume, then diluted back to original volume using lysis buffer to reduce imidazole concentration. Protein concentration was checked using A_280_ and a theoretical ϵ_280_ = 17460M^-1^cm^-1^ The purification tag was removed by adding His-tagged Rhinovirus 3C protease in a 50:1 Avr4-to-protease ratio and 10μM of BME. The cleavage reaction was conducted at 4°C with stirring for 15 hours. The cleaved tag and protease were removed by flowing cleavage product back over the Ni-NTA column. The collected flow-through was checked for purity on SDS gel. *Cf*Avr4 proved to be unamenable to dialysis. Buffer exchange was conducted via Amicon Ultra-15 Centrifugal Filters (Millipore, 3000 NMWL). The recovered protein was concentrated down to <3mL and diluted to 14mL using the crystallization buffer, 10mM Bis-Tris, pH 6.5, 100mM NaCl. This concentration and dilution was repeated twice more before the final concentration step for full buffer exchange.

### *Cf*Avr4 crystallization and data collection

*E*. *coli*-produced *Cf*Avr4 was concentrated to 100mg/mL and mixed with chitin hexasaccharide to reach a final concentration of 80mg/mL and 8mM carbohydrate, a 1:1 stoichiometric ratio. The protein and ligand were co-crystallized by sitting-drop vapor diffusion in 0.1M Tris:HCl, pH 8.5, 30% (w/v) PEG-4000, 0.8M LiCl at 4°C. Crystals were harvested and briefly soaked in reservoir buffer supplemented with 30% ethylene glycol prior to flash-cooling in liquid nitrogen. X-ray diffraction data were collected at ALS beamline 8.3.1. Crystals belong to space group *P*2_1_ with unit cell parameters of: *a* = 39.83 Å, *b* = 41.08 Å, *c* = 121.30 Å, β = 97.871°. A Matthews coefficient [41] was calculated to be 2.14 Å^3^/Da (solvent content = 42.5%) assuming four monomers per asymmetric unit. Phases were determined by molecular replacement using *Pf*Avr4 (PDB: 4Z4A) as a search model. The resulting structure was refined to a resolution of 1.95Å in the space group *P*2_1_. The structure was refined to a *R*_factor_ = 16.70% and an *R*_free_ = 21.45% using Phenix Refine [42]. Data collection and refinement statistics are shown in S1 Table. The refinement restraints for the hexasaccharide of GlcNAc were generated using Phenix eLBOW [43]. Atomic coordinates along with structure factors have been deposited in the Protein Data Bank, PDB ID: 6BN0.

### Site-directed mutagenesis

Primers for the site-directed mutagenesis were designed using the Agilent QuikChange Primer Design tool. Primers were purchased from Integrated DNA Technologies (IDT) and mutagenesis reactions were performed using Accuzyme PCR mix. Reaction products were purified and transformed into BL21 (DE3) cells. Colonies were selected and a Miniprep was performed to amplify the DNA. Collected DNA was sequenced to confirm proper, in-frame mutation (QuintaraBio). Some reactions repeatedly resulted in primer duplications and further experiments were performed using the half-reaction procedure to prevent the duplications. Sequence-confirmed mutations were transformed into the Rosetta-gami B cells for expression.

### Isothermal titration calorimetry

All ITC experiments were performed on a TA Instruments low volume NanoITC with a 186uL reaction cell and a reference cell filled with degassed Milli-Q grade water. Reactions were run in a buffer containing 10mM Bis-Tris, pH 6.5, 100 mM NaCl. The reservoir solution was filled with protein solution and continuously stirred while Chitin hexasaccharide solutions titrated in 2.5μL aliquots for 19 injections, with an initial 1μL injection, for a total injection volume of 48.5μL. The carbohydrate solutions were made by dissolving the dry powder in buffer to a concentration of 20mM (confirmed using methods described above) and then diluted to the working concentration using the final buffer exchange flow through to match the buffers as closely as possible. The integrated heats, after correction for the heat of dilution were analyzed using the NanoAnalyze software from ITC Technologies. An individual binding site model was used to fit the binding isotherm. All experiments were replicated a minimum of three times.

### *In vitro* protection assays of *T*. *viride* germlings against chitinases

The ability of the WT-CfAvr4 and of ChBD mutants to protect against chitinases was evaluated in *in vitro* protection assays conducted as described before [6]. Briefly, spores of *T*. *viride* (3∙10^3^) were pre-germinated overnight at room temperature in 100 μl of half-strength PDB medium in 96 well plates. The following day, 5 μM of WT-CfAvr4 or ChBD mutants were mixed with 7.5 units of Zymolyase (Zymo Research cat. no. E1004) and 0.2 units of bacterial chitinases (Sigma-Aldrich cat. no. C6137) and the mixture was added to the microtitre plate wells containing the pre-germinated spores in a final volume of 150 μl. Plates were incubated at 25°C for 6–8 h before evaluating under the microscope fungal growth and the ability of the proteins to provide protection against chitinases. Zymolase has β-1,3 glucanase and β-1,3-glucan laminaripentaohydrolase activity and is added to the mixture in order to remove the surface glucans from the fungal cell wall and thus expose the underlying chitin layer.

### Protein infiltrations into tomato leaves

Tomato plants of cv Moneymaker (MM), which does not carry *Cf-4* or any other functional *Cf* resistance genes against *C*. *fulvum*, and of cv Purdue 135, which expresses a functional *Cf-4* resistance gene, were grown in a growth chamber with 16 h of artificial light and 70% humidity at 27°C for 6 weeks. Tomato seeds were obtained from the Tomato Genetic Resource Center (TGRC) at UC Davis. WT-*Cf*Avr4 and mutants thereof with single point mutations at selected amino acids were produced and purified from *E*. *coli* Rosetta-gami B cells as description above. Proteins were infiltrated at a concentration of 5 μg/mL and 10 μg/mL into the back side of MM or Purdue 135 tomato leaves using a one mL syringe and the HR response was recorded at 6 days post-infiltrations.

### *Agrobacterium tumefaciens*-mediated transformation assay (ATTA)

The *Agrobacterium tumefaciens* transient transformation assay (ATTA) was used for the transient co-expression into leaves of *Nicotiana benthamiana* of Cf-4 with the WT-*Cf*Avr4 and the mutants thereof tested in this study [6, 44]. Briefly, *N*. *benthamiana* plants were grown in a growth chamber with 16 h of artificial light and 70% humidity, at 25°C for 4 weeks. The true mature form of the WT-CfAvr4 (i.e. between Lys30-Gln115) [3] and of the mutants thereof were singly cloned into the binary expression vector pICH47742 downstream of the PR1A signal sequence of *Nicotiana tabacum* for targeted secretion into the apoplast, and under the control of CaMV 35S promoter and NOS terminator. The tomato Cf-4 was cloned into pMOG800 as descripted before [20]. Binary vector plasmids were transformed into *A*. *tumefaciens* strain GV3101 by electroporation. For the ATTA assay, agrobacteria transformed with pMOG800:Cf-4 was grown in 10mL LB-mannitol medium (LB medium with 10 g/L mannitol) amended with 50 μg/mL kanamycin and 25 μg/mL rifampicin, whereas agrobacteria transformed with pICH47742:WT-*Cf*Avr4 or pICH4774:*Cf*Avr4-mutants were grown in LB-mannitol amended with 100 μg/mL carbenicillin and 25 μg/mL rifampicin. All cultures were incubated at 28°C for 2 days, after which period they were pelleted at 2800*g* for 15 min, re-suspended in MMAi medium (5 g/L Murashige and Skoog basal salts, 20 g/L sucrose, 10 mM MES, and 200 mM acetosyringone) at an optical cell density (A_600_) of 2.0, and further incubated for 2–4 h at room temperature. Agroinfiltrations were performed by mixing agrobacteria containing pMOG800:Cf-4 with agrobacteria containing pICH47742_WT-*Cf*Avr4 or pICH47742:CfAvr4-mutants at three different ratios of optical cell densities, i.e. A_600_0.5:A_600_1.0, A_600_0.5:A_600_0.5, and A_600_0.5:A_600_0.25 of. The mixture of agrobacteria was infiltrated into the leaves of *N*. *benthamiana* plants using a 1 mL syringe and the induction of an HR in the infiltrated leaf sectors was evaluated at 5 days post-infiltrations. Control infiltrations were made using only the pMOG800:Cf-4 transformed agrobacteria and the agrobacteria containing pICH47742:CfAvr4 or the mutants thereof without mixing the two cultures, at cell densities of A_600_0.25, A_600_0.5, and A_600_1.0.

### Subtilisin treatments

The WT-*Cf*Avr4 and selected mutants thereof were exposed to the non-specific protease subtilisin in order to determine their resistance against proteolytic degradation. Treatments with subtilisin were made as previously described with minor modifications [6]. Briefly, 40 μM of the *E*. *coli*-produced *Cf*Avr4 and the selected *Cf*Avr4 mutants were mixed with 10 mM of calcium chloride and 500 ng/μL of subtilisin (Sigma-Aldrich) in a total volume of 15 μL. Samples were incubated for 30 min at room temperature, after which period they were denatured by mixing with 10 mM PMSF and SDS-PAGE loading buffer, and heating at 98°C for 10 min. Digestion products were visualized on an SDS-PAGE after staining with Coomassie blue.

### Accession numbers

The atomic coordinates and structure factors have been deposited in the Protein Data Bank, www.wwpdb.org (PDB ID code 6BN0).

## Supporting information

S1 Table Data collection and refinement statistics for *Cf*Avr4 (PDB ID: 6BN0). (PDF)

S2 Table Amino acids in *Cf*Avr4 that interact with (GlcNAc)_6_ in the dimeric assembly and the effect of mutating these residues on the (GlcNAc)_6_ affinity of the protein and its recognition by the Cf-4 immune receptor. (PDF)

S1 Fig Superposition of *Cf*Avr4 to other members of the CBM14 family. *Cf*Avr4 is shown in purple color along with the associated (GlcNAc)_6_ molecule and some amino acid side chains that interact with it. Only *Cf*Avr4 contains helix H1, along with an extended loop between H1 and β-strand A1 that interacts with the reducing end of (GlcNAc)_6_. *Cf*Avr4 superimposes with (**A**) *Pf*Avr4 (PDB Id: 4Z4A), (**B**) tachycitin (PDB Id: 1DQC), (**C**) Der p 23 (PDB Id: 4ZCE), and (**D**) the ChBD of the human chitotriosidase CHIT1 (PDB Id: 5HBF), at an RMSD of 0.794Å, 2.019 Å, 0.699 Å, and 0.686 Å, over 53, 36, 16, and 38 α-carbons, respectively.
(PNG)

S2 Fig The crystallographic asymmetric unit containing the dimer of dimers. The protein is shown in the cartoon representation and the (GlcNAc)_6_ ligand in the stick representation. Each dimer is structurally equivalent, with Chain A being non-crystallographically symmetry related to Chain C and Chain B related to Chain D. The interface between the two dimer pairs is between chain A and chain C. The interactions occur between the β-hairpin connecting β-strands A1 and A2 and the C-terminal end of both chains. The interactions include two H-bonds between the main chain hydroxyl of Pro32 and the side chain amine of Lys59 of both chains. A chloride ion mediated interaction also exists between the main chain nitrogens of Gly89 of chain A and Cys64 of chain C, as well as the symmetry related Gly89 of chain C and Cys64 of chain A. The chloride ions are shown as small green spheres. These chloride ions were confirmed using an anomalous difference electron density map. The total interface surface area between the two dimers is only 620 Å^2^, suggesting the tetrameric unit is likely crystallographically induced and the biologically relevant assembly is a dimer.
(PNG)

S3 Fig Quality of electron density map clearly defines both (GlcNAc)_6_ molecules. Structure of the *Cf*Avr4 dimer bound to two (GlcNAc)_6_ molecules is shown along with the final 2Fo-Fc electron density map contoured at 1σ. The A subunit is shown in magenta and the B subunit in cyan. The N- and C-terminal ends of the protein are labeled along with the monosaccharide ends with 1 designating the reducing end of the sugar. A single (GlcNAc)_6_ unit buries ~450 Å^2^ of surface area in each *Cf*Avr4 monomer, while ~790 Å^2^ of total surface area is buried across each dimer, including both sugar and protein.
(PNG)

S4 Fig Residues involved in binding (GlcNAc)_6_. Each (GlcNAc)_6_ molecule is illustrated showing the major interactions with protein and the other oligosaccharide. Hydrogen bonds are represented by black dashed lines, van der Waals interactions stacking against the pyranose rings is illustrated by green hashed lines. GlcNAc residues are labeled with the reducing end at the right. Atom names for the GlcNAc are shown for sugar 1 of the A subunit in red.
(PNG)

S5 Fig Comparison of dimeric assemblies between *Cf*Avr4 and *Pf*Avr4. The two A chains of each dimer were superimposed. The disposition of the *Cf*Avr4 B chain (cyan) is shifted out ~6.7Å and rotated ~54° relative to the B subunit of *Pf*Avr4 (light-green). The two (GlcNAc)_6_ molecules seen in the *Cf*Av4 structure are omitted for clarity.
(PNG)

S6 Fig Representative ITC data for the WT protein and all of the mutants tested. For each panel, the top graph is the corrected heat released upon each injection. The bottom graph is the integrated heat binding curve with the independent binding site model fitting. The calculated *K*_d_ for each is shown, other thermodynamic values are in S2 Table. The graphs are color coded to reflect the results. Purple is all of the WT data, green is mutations that had little effect on binding, yellow is mutations that had substantial effects on binding, orange is mutations that abolished binding, and blue is mutations to the NDN motif.
(PNG)

S7 Fig Capacity of the WT-*Cf*Avr4 and selected Chtohexaose-binding domain (ChBD) mutants to protect fungal hyphae from chitinases. The WT-*Cf*Avr4 is able to protect hyphae of *Trichoderma viride* from the hydrolytic activity of chitinases supplemented with basic β-1,3-glucanases, evidenced by the mycelial growth of the fungus beyond that of the BSA control. Mutants W100A and D102A that do not have any detectable affinity for (GlcNAc)_6_ (S6 Fig) fail to protect the fungal hyphae, whereas mutants Q69N and K84A that exhibit reduced affinity for (GlcNAc)_6_ (S6 Fig) enable fungal growth to levels comparable to those of the WT-*Cf*Avr4. These two mutants, however, show signs of osmotic injuries, such as swollen segments and coagulated cytoplasm (pointed by a white arrow), deformations also seen with the BSA control and the W100A and D102A mutants. Images were taken with a Nikon Diaphot inverted tissue culture microscope at a 10x (far left column) and 40x (right three columns) magnification.
(TIF)

S8 Fig Close-up view of ^93^NDN^95^ loop. Asn93 hydrogen-bonds to Asp102, which is essential for (GlcNAc)_6_ binding. Asn93 also interacts with Asn95. Potential hydrogen bonds are shown as yellow dashed lines.
(PNG)

S9 Fig ChBD or (GlcNAc)_6_-binding mutants of *Cf*Avr4 all trigger a Cf-4 mediated hypersensitive response (HR) when present in sufficient amounts into the leaf apoplast. **(A)** Protein infiltrations into tomato leaves of cv Purdue 135 (+ Cf-4) of the WT-*Cf*Avr4 (WT) and of the *Cf*Avr4 mutants M51A, P53A, Y67F, K84A, P87A, N95A, W100A and Y103F results in the elicitation of a strong and equally in intensity necrosis in the infiltrated leaf sectors at both concentrations tested (i.e. 5 and 10 μg/ml). However, mutants Q69N and D102A elicited a weak HR response at infiltrations with 5 μg/ml, whereas mutants N93A and D94A did not elicit an HR at neither 5 μg/ml nor 10 μg/ml. None of the proteins, WT-*Cf*Avr4 or mutants, elicited an HR when infiltrated into tomato leaves of cv Moneymaker (–Cf-4) at 10 μg/ml. Infiltrations with 5 μg/ml and/or 10 μg/ml were performed on both the left- and right-hand side of the leaf, and necrosis was evaluated 5 days post-infiltration. The buffer alone was also used as a control. **(B)** Transient co-expression with Cf-4 of the WT-*Cf*Avr4 (WT) or mutants M51A, P53A, Y67F, Q69N, K84A, P87A, N93A, D94A, N95A, W100A, D102A and Y103F into *Nicotiana benthamiana* leaves using an *Agrobacterium tumefaciens* transient transformation assay (also known as agroinfiltrations), induces in all cases a strong and similar in intensity HR in the infiltrated leaf sectors, indicating that all mutants eventually trigger a Cf-4 mediated HR when present in sufficient amounts into the leaf apoplast. Co-infiltrations were performed at three different cell density ratios between effector and receptor, i.e. 0.5:1 (A_600_0.25:A_600_0.5), 1:1 (A_600_0.5:A_600_0.5), and 2:1 (A_600_1.0:A_600_0.5). Agro-infiltrations of the single proteins alone (bottom two rows of leaves) were used as controls. In all cases, co-infiltrations of Cf-4 with the WT-*Cf*Avr4 were done on the left-hand side of the leaf, whereas co-infiltrations of Cf-4 with one of the mutants was done on the right-hand side of the leaf. Pictures were taken at 7 days post-infiltrations.
(TIF)

S10 Fig Susceptibility to proteolytic degradation of the WT-*Cf*Avr4 and the ChBD mutants K84A and W100A. The susceptibility to proteolytic degradation of the K84A and W100A ChBD mutants, which although they triggered a full and equal in intensity HR as the WT-*Cf*Avr4 (S9 Fig), they nonetheless exhibit less affinity for (GlcNAc)_6_ (S6 Fig) was examined. Treatment of the WT-*Cf*Avr4 and of the K84A and W100A mutants with 500 ng/μl subtilisin, digests the original full-length protein (blue arrows) to a smaller product that corresponds to the true mature form of *Cf*Avr4 (red arrows). The treatments show that the two mutants are as resilient to proteolysis as the WT-*Cf*Avr4, evidenced by the equal in intensity band corresponding to the mature *Cf*Avr4.
(TIF)

S11 Fig Structures of α-chitin and β-chitin (green and yellow respectively) as determined by X-ray crystallography [34, 35, 45]. α-chitin has an antiparallel arrangement of saccharides such that each polymer alternates between reducing end at bottom, top, bottom. β-chitin has all the polymers parallel. Shown is only two stacked sheets of polymers. For comparison, the two stacked (GlcNAc)_6_ molecules found in the *Cf*Avr4 crystal structure is shown in salmon color, which is similar to the repeating unit in both α- and β-chitin.
(PNG)

S12 Fig A representative calibration curve for the determination of the chitin hexasaccharide concentration. Three replicates were measured at each standard and a linear regression was fit (blue line). The gray bar represents the 95% confidence interval. The fit had an R^2^ value of 0.9739.
(PNG)

S1 Movie Movie showing all four monomers (two dimers) found in the crystallographic asymmetric unit. Each monomer is shown in a different color with the corresponding chitin hexasaccharide drawn as sticks with the same color carbon atoms. The two chloride atoms, identified by anomalous difference electron density, are shown as green spheres.
(MOV)

S2 Movie Movie showing the *Cf*Avr4 dimer binding two (GlcNAc)_6_ saccharides. The A subunit is colored dark gray and the B subunit in light gray. Residues that were tested for binding effects by site directed mutagenesis and ITC are drawn as sticks. Residues that do not substantially effect binding are in slate blue, residues significantly effecting binding are in yellow, and residues that abolish binding are in orange. Hydrogen bonds are shown in yellow dashed lines. Dashes extending to main chain, hydrogen bond to main chain atoms.
(MOV)
