# Supplementary material for: Structure of the Cladosporium fulvum Avr4 effector in complex with (GlcNAc)6 reveals the ligand-binding mechanism and uncouples its intrinsic function from recognition by the Cf-4 resistance protein
Source: PLoS Pathog. 2018 Aug 27;14(8):e1007263. doi: 10.1371/journal.ppat.1007263 (PMC6128652; doi:10.1371/journal.ppat.1007263)
Supplement: S1 Table — (PDF) [file ppat.1007263.s001.pdf]

Supporting Table 1. Data Collection and Refinement Statistics for *Cf*Avr4 (PDB ID: 6BN0).

**X-ray Source**	**ALS 8.3.1**
Wavelength (Å)	1.11583
Temperature (K)	100
Space group	$P2_1$
Unit-cell parameters (Å, °)	a=39.86, b=41.08, c=121.36
	α=γ=90, β=97.87
Resolution (Å)	60.11-1.95 (2.00-1.95)
R_{merge}[a] (%)	5.4 (61.2)
<I/σ(I)>	13.69 (2.12)
$CC_{1/2}$ (%)	99.9 (74.8)
No. of reflections	104,460 (7,850)
No. of unique reflections	28,654 (2,110)
Completeness (%)	99.6 (99.8)
Redundancy	3.65 (3.72)
**Refinement Statistics**	
Resolution (Å)	60.11-1.95 (2.02-1.95)
No. of reflections (F>0) used in refinement	28,641 (2,719)
R_{factor}[b] (%)	16.77
R_{free}[c] (%)	21.40
RMS bond length (Å)	0.013
RMS bond angle (°)	1.261
Overall B Value (Å^2)	44.01
**Ramachandran Plot Statistics**[d]	
Residues	207
Most favored Region	97.7%
Allowed Region	2.3%
Disallowed	0.0%

[a] $R_{merge} = [\sum_h \sum_i |I_h - I_{hi}| / \sum_h \sum_i I_{hi}]$ where I_h is the mean of I_{hi} observations of reflection *h*. Numbers in parenthesis represent highest resolution shell. [b] R_{factor} and [c] $R_{free} = \sum||F_{obs}| - |F_{calc}|| / \sum|F_{obs}|$ x 100 for 95% of recorded data (R_{factor}) or 5% data (R_{free}). [d] From MolProbity (Chen et al., 2010)
