# Supplementary material for: Structure of the Cladosporium fulvum Avr4 effector in complex with (GlcNAc)6 reveals the ligand-binding mechanism and uncouples its intrinsic function from recognition by the Cf-4 resistance protein
Source: PLoS Pathog. 2018 Aug 27;14(8):e1007263. doi: 10.1371/journal.ppat.1007263 (PMC6128652; doi:10.1371/journal.ppat.1007263)
Supplement: S2 Table — (PDF) [file ppat.1007263.s002.pdf]

**Supporting Table 2.** Amino acids in *Cf*Avr4 that interact with (GlcNAc)$_6$ in the dimeric assembly and the effect of mutating these residues on the (GlcNAc)$_6$ affinity of the protein and its recognition by the Cf-4 immune receptor.

| **Amino acids interacting with (GlcNAc)$_6$ as determined by the solution structure of the *Cf*Avr4-(GlcNAc)$_6$ complex** | | **Thermodynamic binding parameters to (GlcNAc)$_6$ as determined by isothermal titration calorimetry (ITC)** | | | | | | **Protein stability and recognition by Cf-4 as determined by pure protein infiltrations and ATTAs** | | |
|---|---|---|---|---|---|---|---|---|---|---|
| **Amino acid (Chain)** | **Interaction with GlcNAc unit in (GlcNAc)$_6$ (Chain)** | **Introduced mutation** | **Protein (mM)** | **(GlcNAc)$_6$ Ligand (mM)** | **$K_d$ (μM)** | **n** | **$\Delta H$ (kJ/mol)** | **Recognition by Cf-4 (pure protein infiltr)** | **Recognition by Cf-4 (ATTAs)** | **Stability (Subtilisin assay)** |
| WT-CfAvr4 | (GlcNAc)$_6$ | NA | 0.198 | 2.10 | 7.63 ± 1.74 | 1.17 ± 0.11 | -40.87 ± 1.42 | Y / Y[1] | Y / Y / Y [2] | S3 |
| WT-CfAvr4 w/o tag | (GlcNAc)$_6$ | NA | 0.333 | 2.05 | 6.73 ± 1.49 | 1.09 ± 0.12 | -37.58 ± 3.51 | - | - | - |
| WT-CfAvr4 reverse titration | (GlcNAc)$_6$ | NA | 1.03 | 0.33 | 7.16 ± 0.76 | 1.01 ± 0.06 | -41.97 ± 1.90 | NA | NA | NA |
| Lys49 (Chain A) | GlcNAc-1 (Chain A) | - | - | - | - | - | - | - | - | - |
| Cys50 (Chain A) | GlcNAc-2 (Chain A) | - | - | - | - | - | - | - | - | |
| Met51 (Chain A) | GlcNAc-2 (Chain A) | M51A | 0.293 | 1.56 | 9.20 ± 1.47 | 1.01 ± 0.08 | -33.30 ± 0.87 | Y / Y | Y / Y / Y | |
| Pro53 (Chain A) | GlcNAc-1 (Chain A) | P53A | 0.300 | 1.52 | 11.34 ± 2.58 | 0.94 ± 0.04 | -40.86 ± 2.26 | Y / Y | Y / Y / Y | |
| Tyr67 (Chain A)<br>Tyr67 (Chain B) | GlcNAc-2 (Chain B)<br>GlcNAc-4 (Chain A) | Y67F | 0.201 | 2.08 | 6.86 ± 1.24 | 1.01 ± 0.09 | -27.70 ± 1.01 | Y / Y | Y / Y / Y | |
| Gln69 (Chain A)<br><br>Gln69 (Chain B) | GlcNAc-2 (Chain A)<br>GlcNAc-1 (Chain B)<br>GlcNAc-2 (Chain B)<br>GlcNAc-3 (Chain A) | Q69N | 0.304 | 2.98 | 130.95 ± 31.47 | 1.01 ± 0.10 | -18.84 ± 4.38 | P / Y | Y / Y / Y | PU |
| Lys84 (Chain A)<br>Lys84 (Chain B) | GlcNAc-1 (Chain B)<br>GlcNAc-3 (Chain A) | K84A | 0.598 | 3.11 | 120.67 ± 18.77 | 0.92 ± 0.11 | -19.53 ± 1.73 | Y / Y | Y / Y / Y | S |
| Pro87 (Chain A)<br>Pro87 (Chain A)<br>Pro87 (Chain B)<br>Pro87 (Chain B) | GlcNAc-3 (Chain B)<br>GlcNAc-4 (Chain B)<br>GlcNAc-5 (Chain A)<br>GlcNAc-6 (Chain A) | P87A | 0.298 | 1.04 | 17.94 ± 0.44 | 0.91 ± 0.03 | -41.82 ± 1.94 | Y / Y | Y / Y / Y | |
| Trp100 (Chain A) | GlcNAc-5 (Chain A) | W100A | 0.300 | no binding | no binding | no binding | no binding | Y / Y | Y / Y / Y | S |
| Cys101 (Chain A) | GlcNAc-5 (Chain A) | - | - | - | - | - | - | - | - | - |
| Asp102 (Chain A) | GlcNAc-6 (Chain B) | D102A | 0.295 | no binding | no binding | no binding | no binding | P / Y | Y / Y / Y | PU |
| Tyr103 (Chain A) | GlcNAc-6 (Chain A)<br>GlcNAc-5 (Chain B) | Y103F | 0.310 | 2.10 | 12.19 ± 4.50 | 1.01 ± 0.07 | -32.32 ± 0.50 | Y / Y | Y / Y / Y | |
| Asn93 (Chain A/B) | None | N93A | 0.240 | 2.43 | 44.70 ± 2.74 | 0.95 ± 0.12 | -8.78 ± 0.98 | N / N | Y / Y / Y | PU |
| Asp94 (Chain A/B | None | D94A | 0.213 | 2.26 | 37.63 ± 7.22 | 1.08 ± 0.07 | -12.83 ± 0.75 | N / N | Y / Y / Y | PU |
| Asn95 (Chain A/B | None | N95A | 0.206 | 1.10 | 18.85 ± 6.03 | 1.06 ± 0.03 | -25.20 ± 0.87 | Y / Y | Y / Y / Y | |

[1] Infiltrations with 5 μg/ml / 10 μg/ml. Y: Yes HR; P: Partial HR; N: No HR; [2] Co-infiltrations between effector and receptor of at cell density ratios of 0.5:1 / 1:1 / 2:1. Y: Yes HR; [3] S: stable; PU: partially unstable
