# Supplementary figures and images for: Structure of the Cladosporium fulvum Avr4 effector in complex with (GlcNAc)6 reveals the ligand-binding mechanism and uncouples its intrinsic function from recognition by the Cf-4 resistance protein
Source: PLoS Pathog. 2018 Aug 27;14(8):e1007263. doi: 10.1371/journal.ppat.1007263 (PMC6128652; doi:10.1371/journal.ppat.1007263)

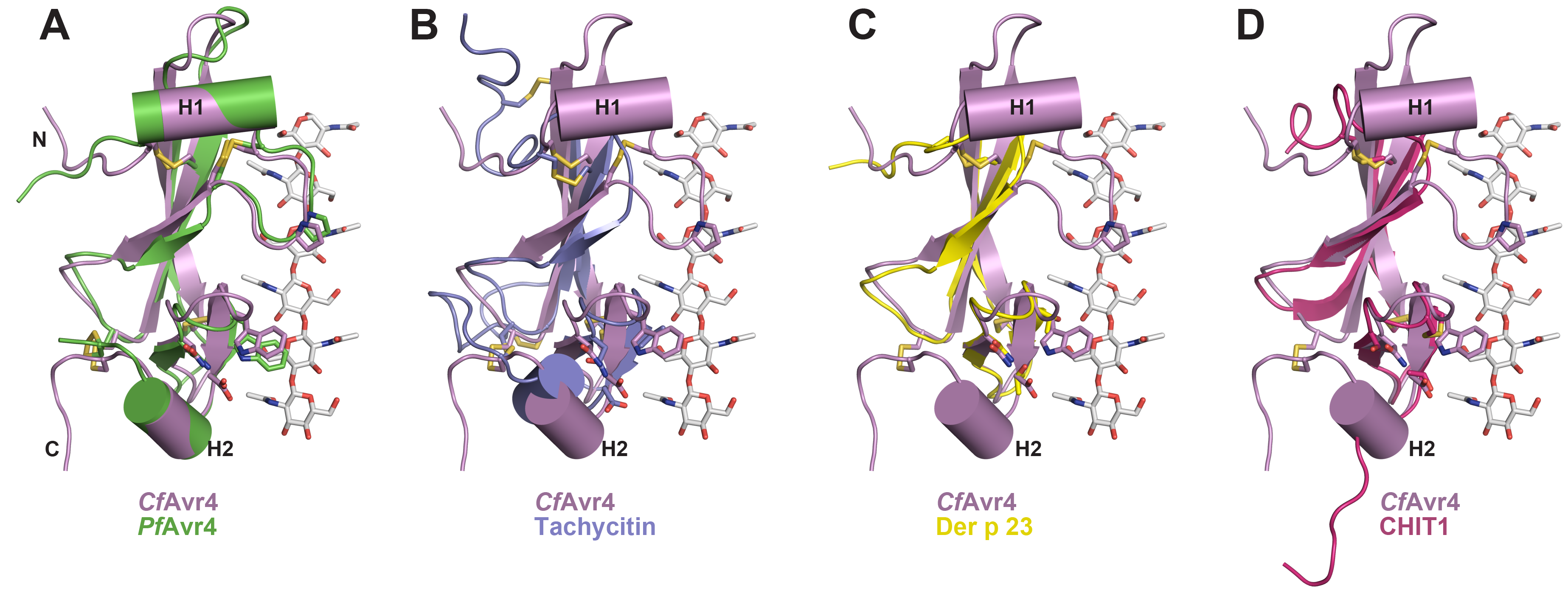

Supplement: S1 Fig — CfAvr4 is shown in purple color along with the associated (GlcNAc)6 molecule and some amino acid side chains that interact with it. Only CfAvr4 contains helix H1, along with an extended loop between H1 and β-strand A1 that interacts with the reducing end of (GlcNAc)6. CfAvr4 superimposes with (A) PfAvr4 (PDB Id: 4Z4A), (B) tachycitin (PDB Id: 1DQC), (C) Der p 23 (PDB Id: 4ZCE), and (D) the ChBD of the human chitotriosidase CHIT1 (PDB Id: 5HBF), at an RMSD of 0.794Å, 2.019 Å, 0.699 Å, and 0.686 Å, over 53, 36, 16, and 38 α-carbons, respectively. (PNG) [file ppat.1007263.s003.png]

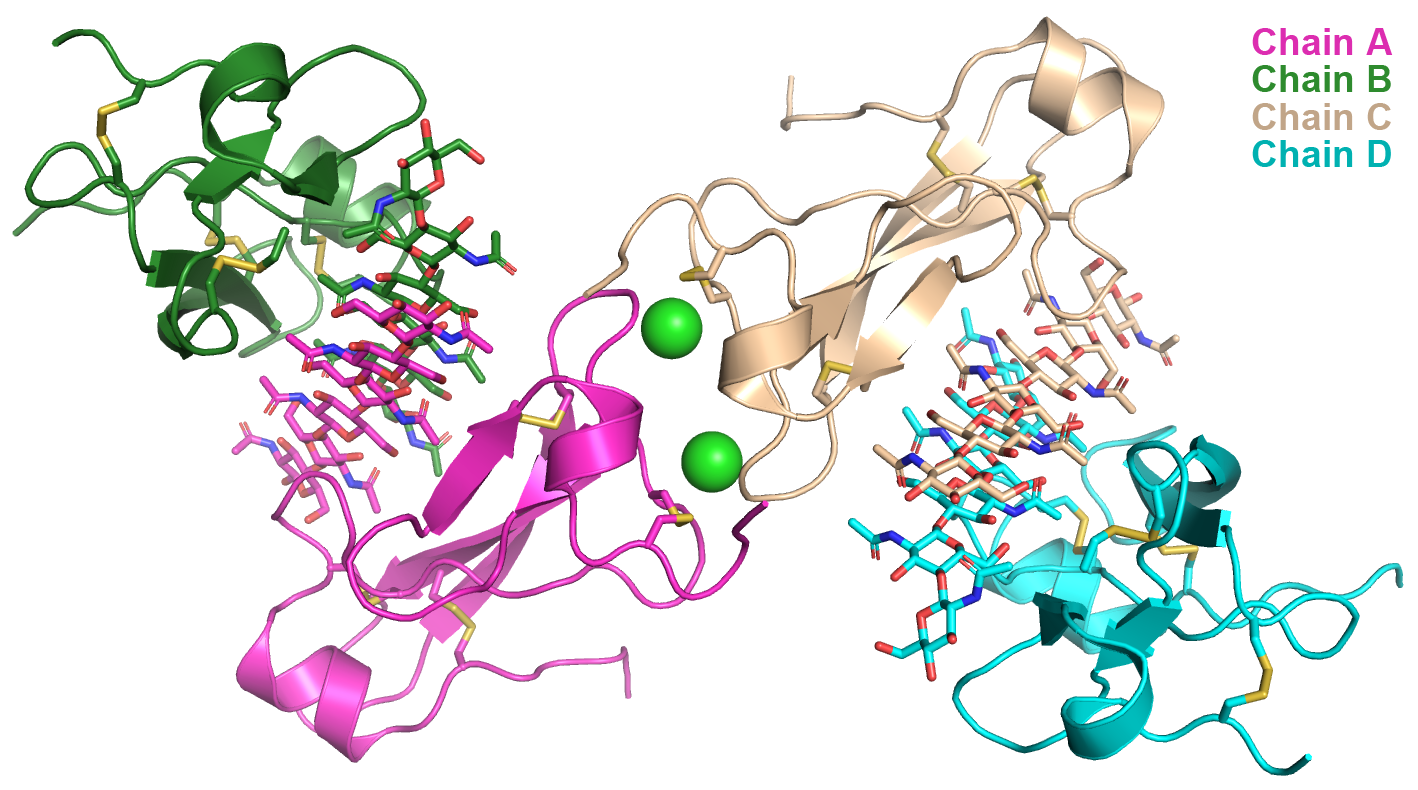

Supplement: S2 Fig — The protein is shown in the cartoon representation and the (GlcNAc)6 ligand in the stick representation. Each dimer is structurally equivalent, with Chain A being non-crystallographically symmetry related to Chain C and Chain B related to Chain D. The interface between the two dimer pairs is between chain A and chain C. The interactions occur between the β-hairpin connecting β-strands A1 and A2 and the C-terminal end of both chains. The interactions include two H-bonds between the main chain hydroxyl of Pro32 and the side chain amine of Lys59 of both chains. A chloride ion mediated interaction also exists between the main chain nitrogens of Gly89 of chain A and Cys64 of chain C, as well as the symmetry related Gly89 of chain C and Cys64 of chain A. The chloride ions are shown as small green spheres. These chloride ions were confirmed using an anomalous difference electron density map. The total interface surface area between the two dimers is only 620 Å2, suggesting the tetrameric unit is likely crystallographically induced and the biologically relevant assembly is a dimer. (PNG) [file ppat.1007263.s004.png]

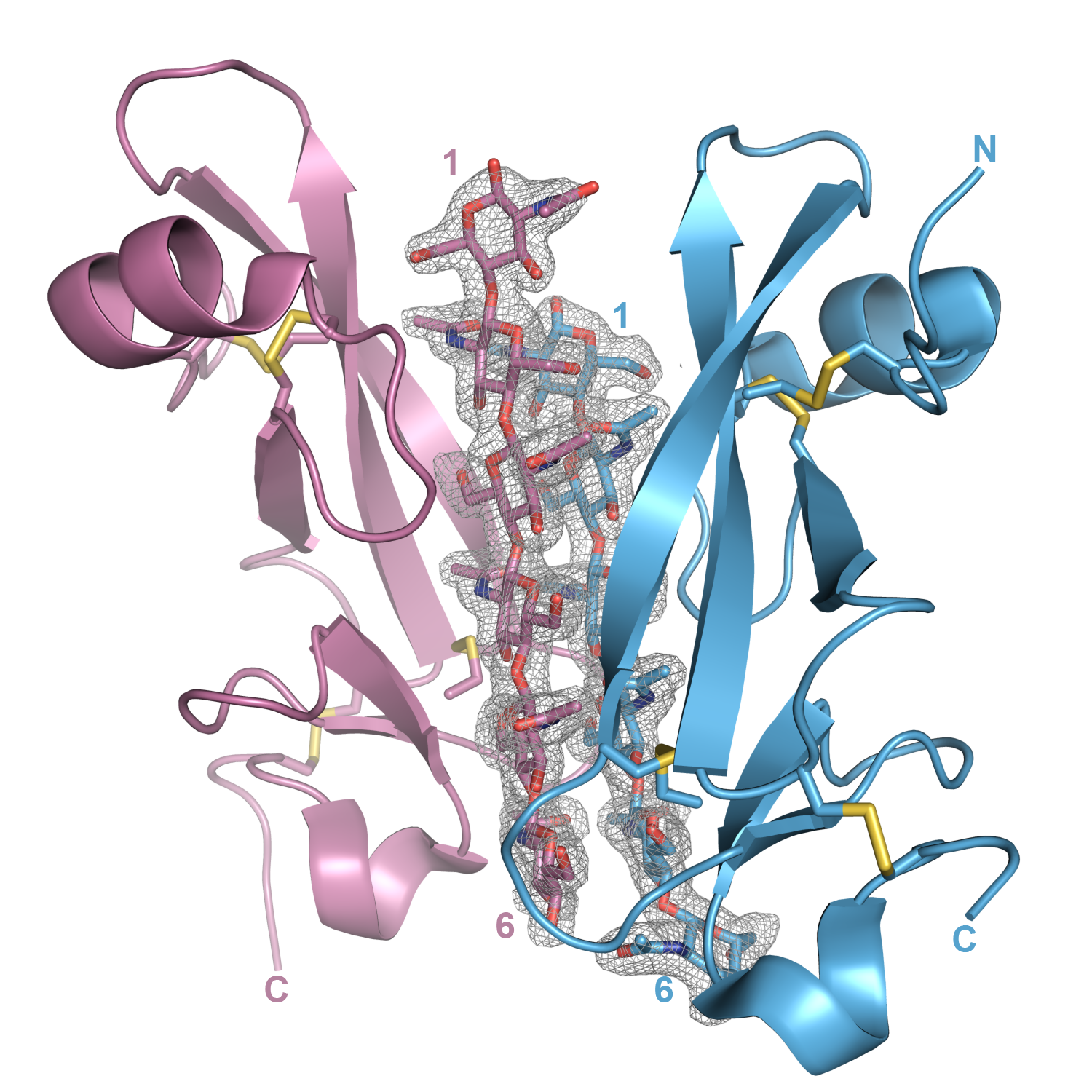

Supplement: S3 Fig — Structure of the CfAvr4 dimer bound to two (GlcNAc)6 molecules is shown along with the final 2Fo-Fc electron density map contoured at 1σ. The A subunit is shown in magenta and the B subunit in cyan. The N- and C-terminal ends of the protein are labeled along with the monosaccharide ends with 1 designating the reducing end of the sugar. A single (GlcNAc)6 unit buries ~450 Å2 of surface area in each CfAvr4 monomer, while ~790 Å2 of total surface area is buried across each dimer, including both sugar and protein. (PNG) [file ppat.1007263.s005.png]

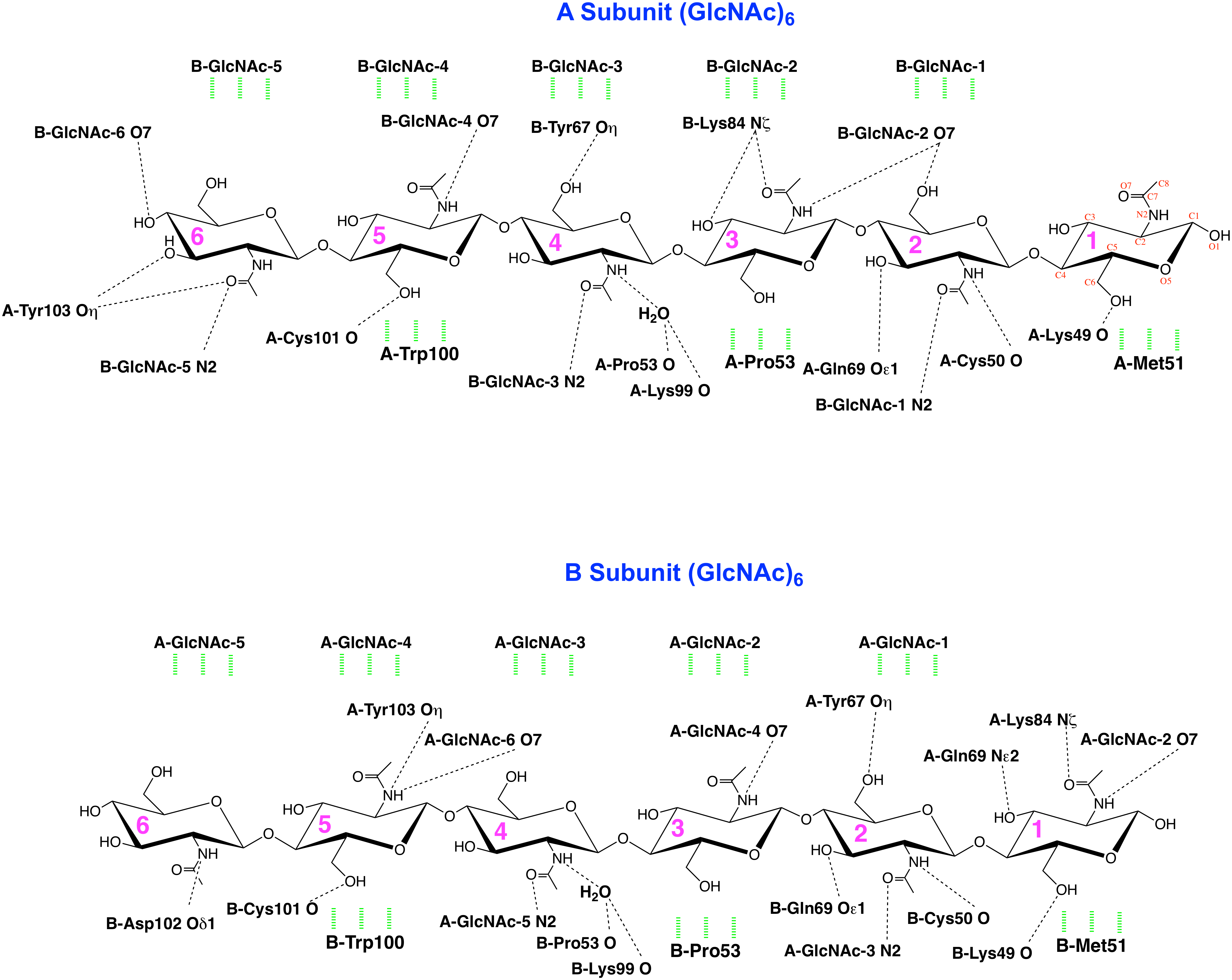

Supplement: S4 Fig — Each (GlcNAc)6 molecule is illustrated showing the major interactions with protein and the other oligosaccharide. Hydrogen bonds are represented by black dashed lines, van der Waals interactions stacking against the pyranose rings is illustrated by green hashed lines. GlcNAc residues are labeled with the reducing end at the right. Atom names for the GlcNAc are shown for sugar 1 of the A subunit in red. (PNG) [file ppat.1007263.s006.png]

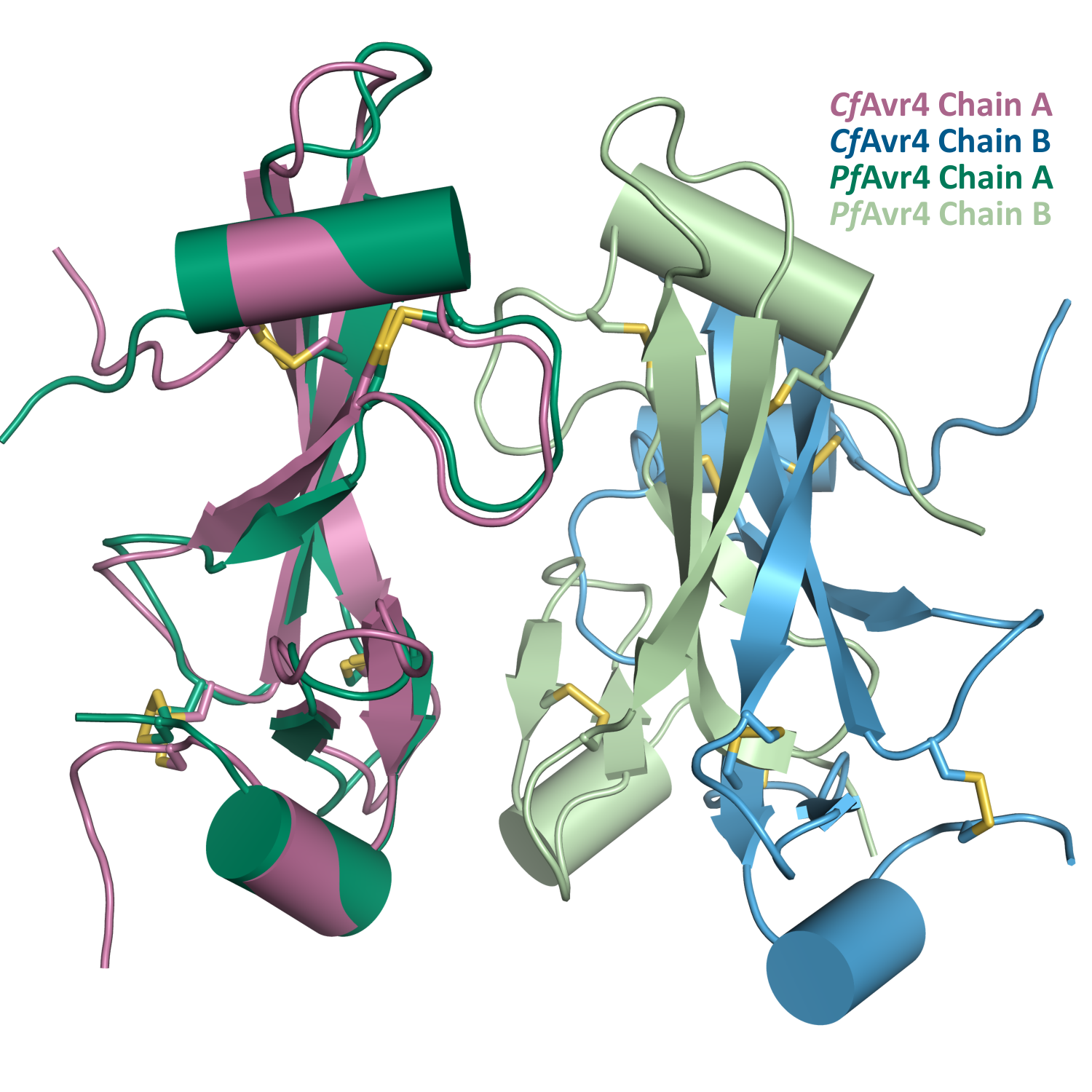

Supplement: S5 Fig — The two A chains of each dimer were superimposed. The disposition of the CfAvr4 B chain (cyan) is shifted out ~6.7Å and rotated ~54° relative to the B subunit of PfAvr4 (light-green). The two (GlcNAc)6 molecules seen in the CfAv4 structure are omitted for clarity. (PNG) [file ppat.1007263.s007.png]

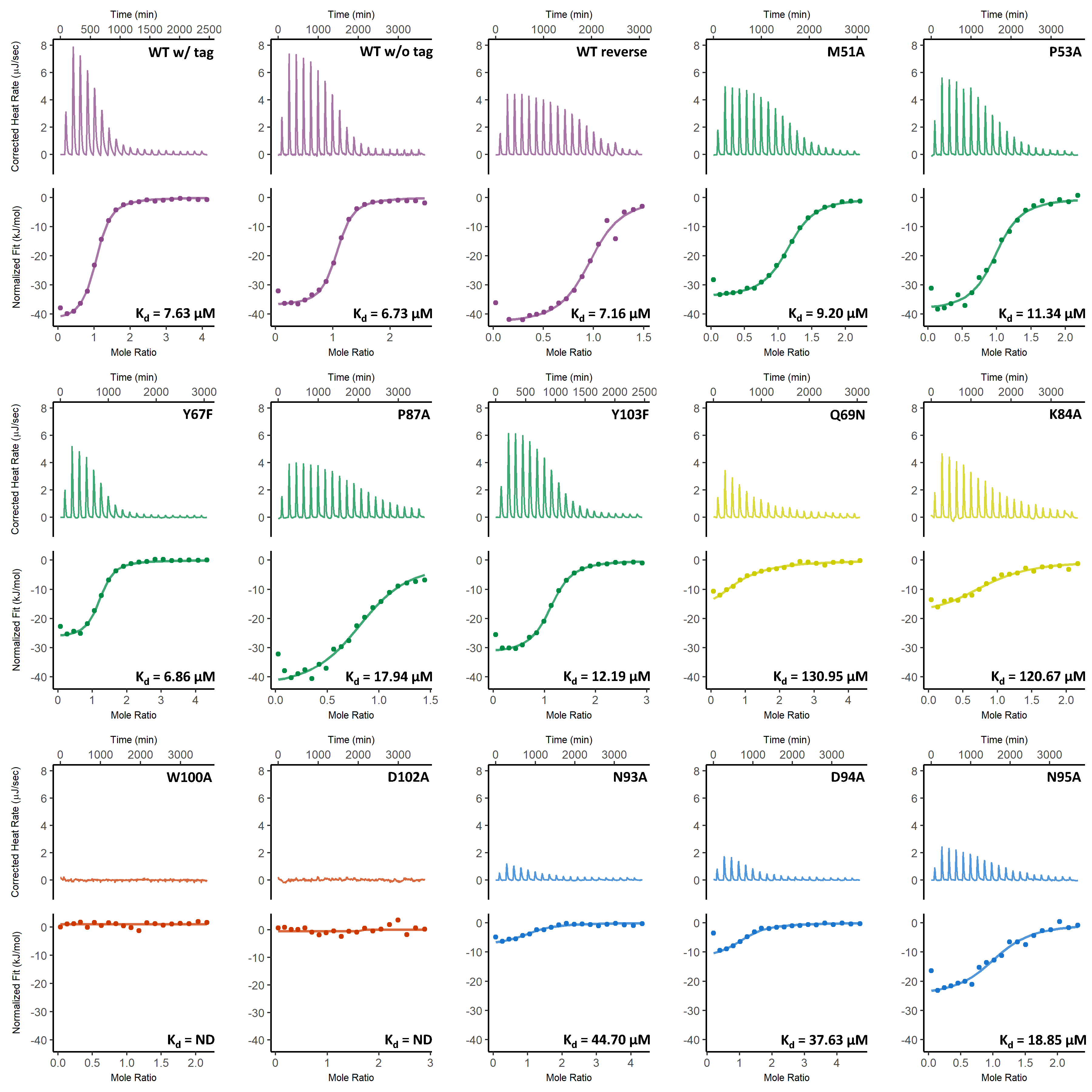

Supplement: S6 Fig — For each panel, the top graph is the corrected heat released upon each injection. The bottom graph is the integrated heat binding curve with the independent binding site model fitting. The calculated Kd for each is shown, other thermodynamic values are in S2 Table. The graphs are color coded to reflect the results. Purple is all of the WT data, green is mutations that had little effect on binding, yellow is mutations that had substantial effects on binding, orange is mutations that abolished binding, and blue is mutations to the NDN motif. (PNG) [file ppat.1007263.s008.png]

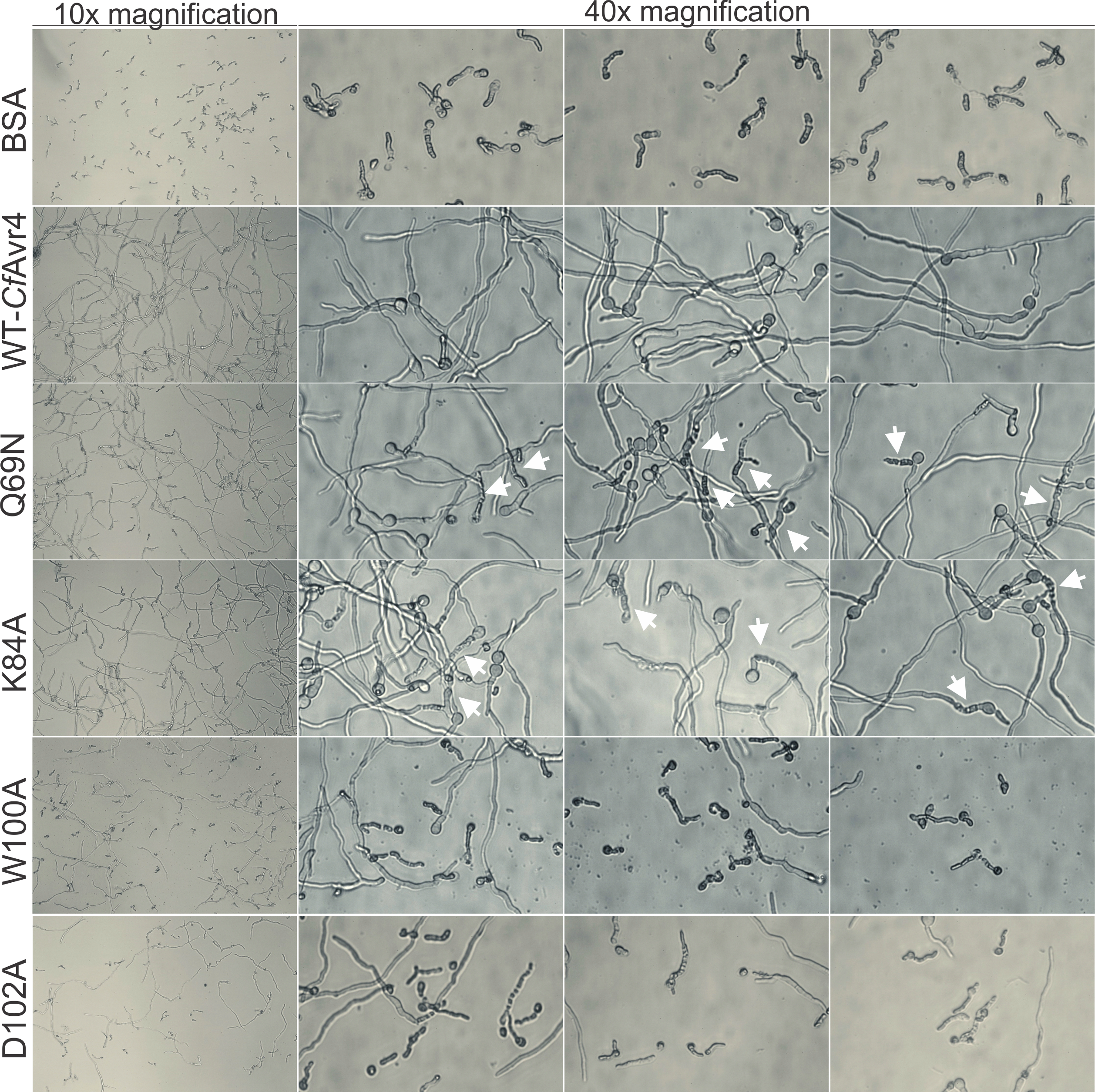

Supplement: S7 Fig — The WT-CfAvr4 is able to protect hyphae of Trichoderma viride from the hydrolytic activity of chitinases supplemented with basic β-1,3-glucanases, evidenced by the mycelial growth of the fungus beyond that of the BSA control. Mutants W100A and D102A that do not have any detectable affinity for (GlcNAc)6 (S6 Fig) fail to protect the fungal hyphae, whereas mutants Q69N and K84A that exhibit reduced affinity for (GlcNAc)6 (S6 Fig) enable fungal growth to levels comparable to those of the WT-CfAvr4. These two mutants, however, show signs of osmotic injuries, such as swollen segments and coagulated cytoplasm (pointed by a white arrow), deformations also seen with the BSA control and the W100A and D102A mutants. Images were taken with a Nikon Diaphot inverted tissue culture microscope at a 10x (far left column) and 40x (right three columns) magnification. (TIF) [file ppat.1007263.s009.tif]

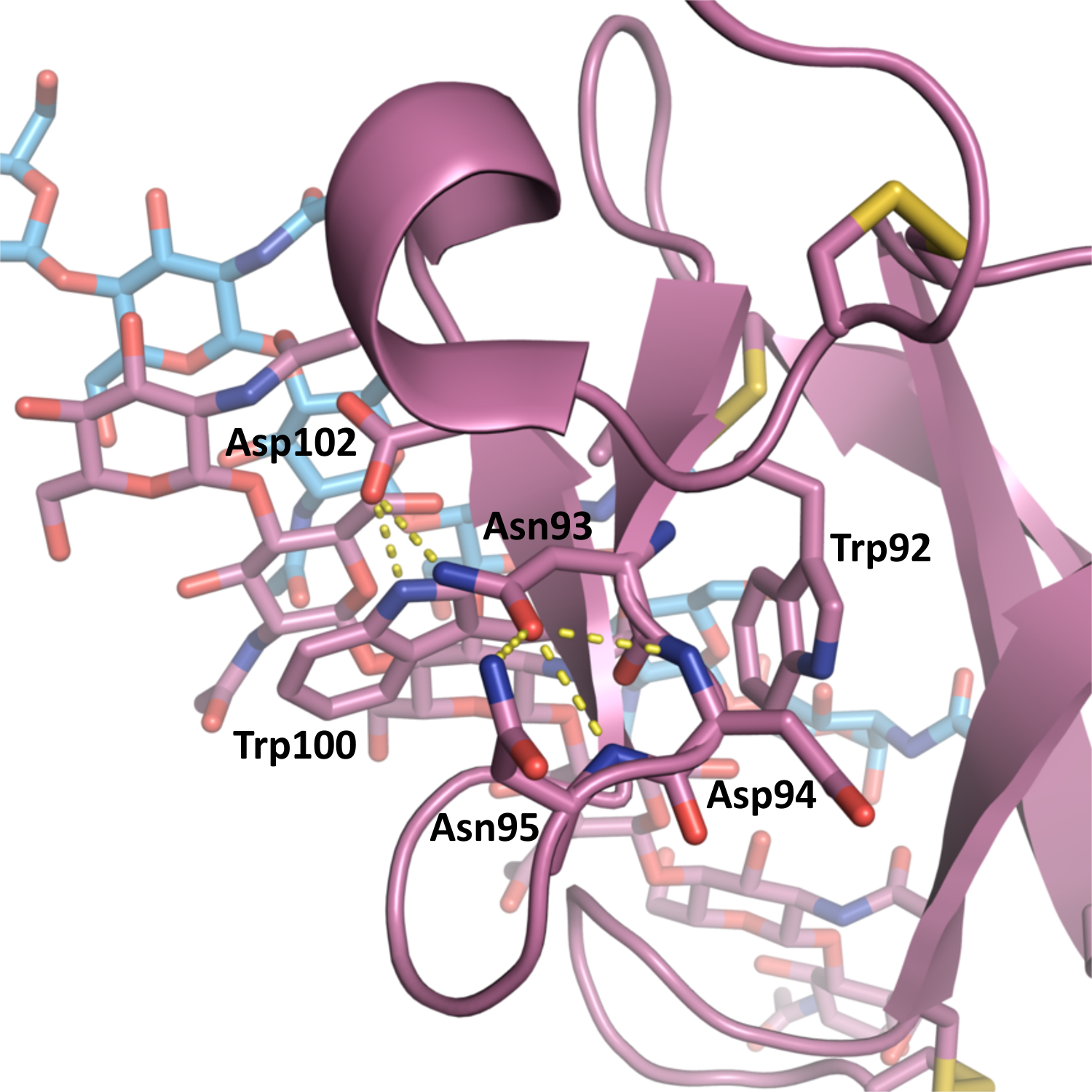

Supplement: S8 Fig — Asn93 hydrogen-bonds to Asp102, which is essential for (GlcNAc)6 binding. Asn93 also interacts with Asn95. Potential hydrogen bonds are shown as yellow dashed lines. (PNG) [file ppat.1007263.s010.png]

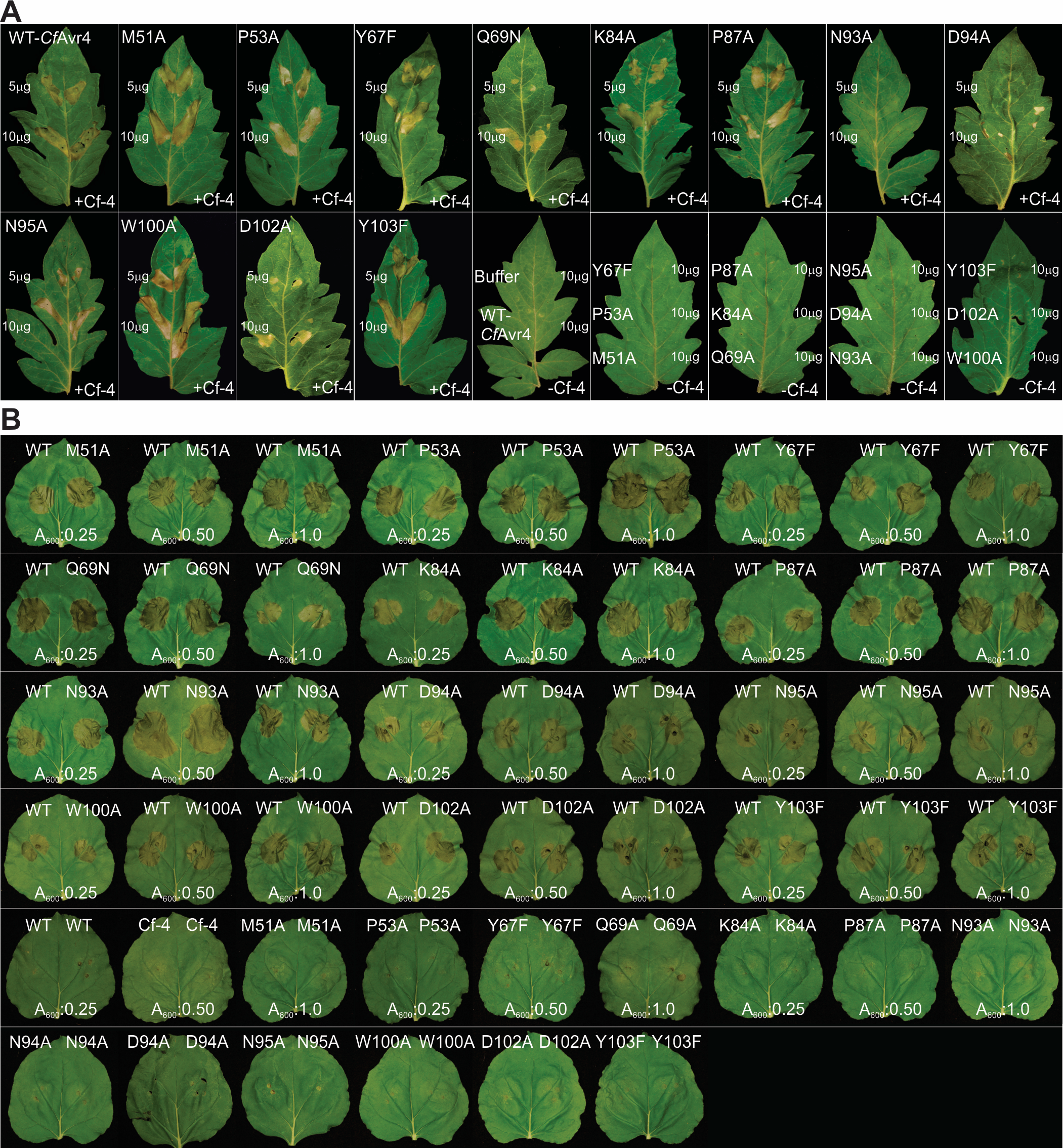

Supplement: S9 Fig — (A) Protein infiltrations into tomato leaves of cv Purdue 135 (+ Cf-4) of the WT-CfAvr4 (WT) and of the CfAvr4 mutants M51A, P53A, Y67F, K84A, P87A, N95A, W100A and Y103F results in the elicitation of a strong and equally in intensity necrosis in the infiltrated leaf sectors at both concentrations tested (i.e. 5 and 10 μg/ml). However, mutants Q69N and D102A elicited a weak HR response at infiltrations with 5 μg/ml, whereas mutants N93A and D94A did not elicit an HR at neither 5 μg/ml nor 10 μg/ml. None of the proteins, WT-CfAvr4 or mutants, elicited an HR when infiltrated into tomato leaves of cv Moneymaker (–Cf-4) at 10 μg/ml. Infiltrations with 5 μg/ml and/or 10 μg/ml were performed on both the left- and right-hand side of the leaf, and necrosis was evaluated 5 days post-infiltration. The buffer alone was also used as a control. (B) Transient co-expression with Cf-4 of the WT-CfAvr4 (WT) or mutants M51A, P53A, Y67F, Q69N, K84A, P87A, N93A, D94A, N95A, W100A, D102A and Y103F into Nicotiana benthamiana leaves using an Agrobacterium tumefaciens transient transformation assay (also known as agroinfiltrations), induces in all cases a strong and similar in intensity HR in the infiltrated leaf sectors, indicating that all mutants eventually trigger a Cf-4 mediated HR when present in sufficient amounts into the leaf apoplast. Co-infiltrations were performed at three different cell density ratios between effector and receptor, i.e. 0.5:1 (A6000.25:A6000.5), 1:1 (A6000.5:A6000.5), and 2:1 (A6001.0:A6000.5). Agro-infiltrations of the single proteins alone (bottom two rows of leaves) were used as controls. In all cases, co-infiltrations of Cf-4 with the WT-CfAvr4 were done on the left-hand side of the leaf, whereas co-infiltrations of Cf-4 with one of the mutants was done on the right-hand side of the leaf. Pictures were taken at 7 days post-infiltrations. (TIF) [file ppat.1007263.s011.tif]

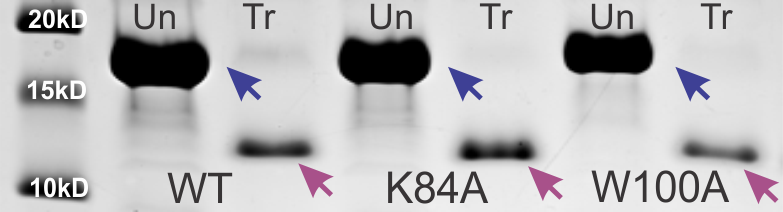

Supplement: S10 Fig — The susceptibility to proteolytic degradation of the K84A and W100A ChBD mutants, which although they triggered a full and equal in intensity HR as the WT-CfAvr4 (S9 Fig), they nonetheless exhibit less affinity for (GlcNAc)6 (S6 Fig) was examined. Treatment of the WT-CfAvr4 and of the K84A and W100A mutants with 500 ng/μl subtilisin, digests the original full-length protein (blue arrows) to a smaller product that corresponds to the true mature form of CfAvr4 (red arrows). The treatments show that the two mutants are as resilient to proteolysis as the WT-CfAvr4, evidenced by the equal in intensity band corresponding to the mature CfAvr4. (TIF) [file ppat.1007263.s012.tif]

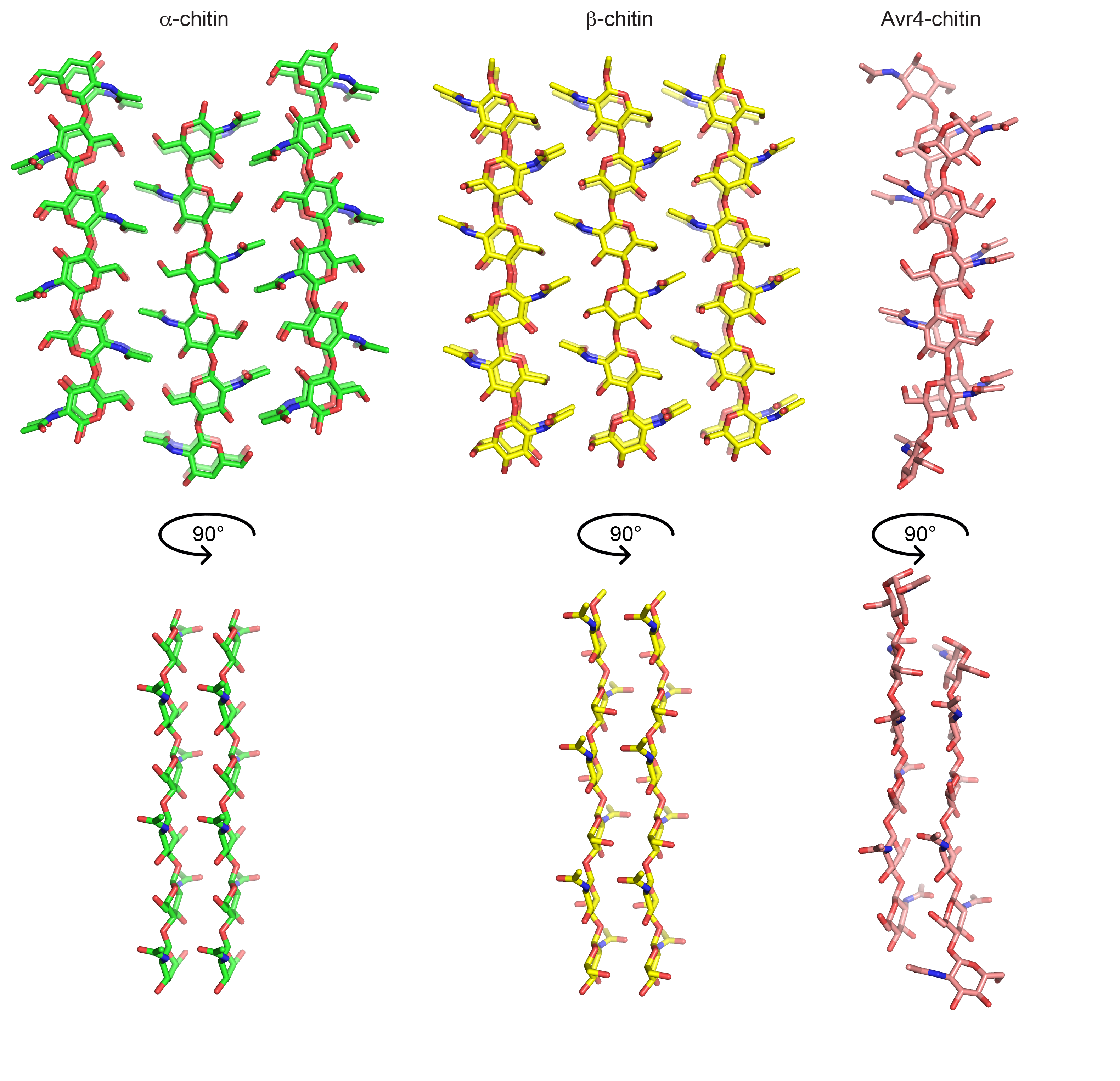

Supplement: S11 Fig — α-chitin has an antiparallel arrangement of saccharides such that each polymer alternates between reducing end at bottom, top, bottom. β-chitin has all the polymers parallel. Shown is only two stacked sheets of polymers. For comparison, the two stacked (GlcNAc)6 molecules found in the CfAvr4 crystal structure is shown in salmon color, which is similar to the repeating unit in both α- and β-chitin. (PNG) [file ppat.1007263.s013.png]

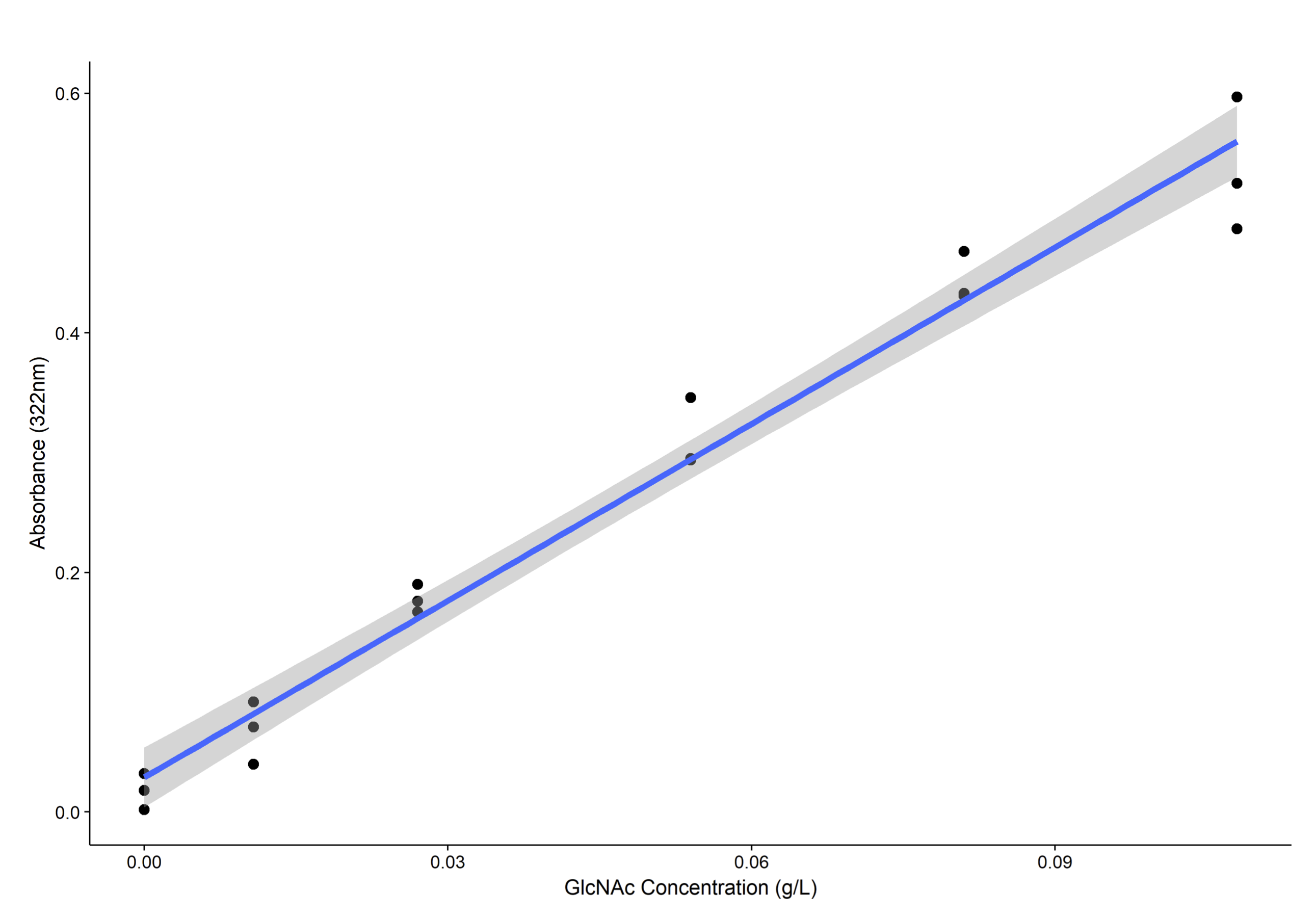

Supplement: S12 Fig — Three replicates were measured at each standard and a linear regression was fit (blue line). The gray bar represents the 95% confidence interval. The fit had an R2 value of 0.9739. (PNG) [file ppat.1007263.s014.png]
